# Overexpression of a novel peanut NBS‐LRR gene *AhRRS5* enhances disease resistance to *Ralstonia solanacearum* in tobacco

**DOI:** 10.1111/pbi.12589

**Published:** 2016-07-26

**Authors:** Chong Zhang, Hua Chen, Tiecheng Cai, Ye Deng, Ruirong Zhuang, Ning Zhang, Yuanhuan Zeng, Yixiong Zheng, Ronghua Tang, Ronglong Pan, Weijian Zhuang

**Affiliations:** ^1^ College of Plant Protection Fujian Agriculture and Forestry University Fuzhou China; ^2^ Fujian Key Laboratory of Crop Molecular and Cell Biology Fujian Agriculture and Forestry University Fuzhou Fujian China; ^3^ College of Agronomy Zhongkai Agriculture and Engineering College Guangzhou Guangdong China; ^4^ Cash Crops Research Institute Guangxi Academy of Agricultural Sciences Nanning China; ^5^ Department of Life Science and Institute of Bioinformatics and Structural Biology College of Life Science National Tsing Hua University Hsinchu Taiwan

**Keywords:** *Arachis hypogaea*, resistance gene, bacterial wilt, signal transduction, NPR1, tobacco

## Abstract

Bacterial wilt caused by *Ralstonia solanacearum* is a ruinous soilborne disease affecting more than 450 plant species. Efficient control methods for this disease remain unavailable to date. This study characterized a novel nucleotide‐binding site‐leucine‐rich repeat resistance gene *AhRRS5* from peanut, which was up‐regulated in both resistant and susceptible peanut cultivars in response to *R. solanacearum*. The product of *AhRRS5* was localized in the nucleus. Furthermore, treatment with phytohormones such as salicylic acid (SA), abscisic acid (ABA), methyl jasmonate (MeJA) and ethephon (ET) increased the transcript level of *AhRRS5* with diverse responses between resistant and susceptible peanuts. Abiotic stresses such as drought and cold conditions also changed *AhRRS5* expression. Moreover, transient overexpression induced hypersensitive response in *Nicotiana benthamiana*. Overexpression of *AhRRS5* significantly enhanced the resistance of heterogeneous tobacco to *R. solanacearum*, with diverse resistance levels in different transgenic lines. Several defence‐responsive marker genes in hypersensitive response, including SA, JA and ET signals, were considerably up‐regulated in the transgenic lines as compared with the wild type inoculated with *R. solanacearum*. Nonexpressor of pathogenesis‐related gene 1 (*NPR1*) and non‐race‐specific disease resistance 1 were also up‐regulated in response to the pathogen. These results indicate that *AhRRS5* participates in the defence response to *R. solanacearum* through the crosstalk of multiple signalling pathways and the involvement of *NPR1* and R gene signals for its resistance. This study may guide the resistance enhancement of peanut and other economic crops to bacterial wilt disease.

## Introduction

Bacterial wilt caused by *Ralstonia solanacearum* is a destructive soilborne bacterial disease in plants, including peanut (Arachis hypogaea L.), worldwide (Wicker *et al*., 2007). This disease is the key limiting factor for the production yield and quality of peanut, an important oil and food crop in China and the world (Yu *et al*., 2011). *R. solanacearum* infects more than 450 plant species, including many important crops, such as peanut, tomato, tobacco, potato, pepper, soybean and rape. However, effective techniques to control this disease remain unavailable to date (Gururani *et al*., 2012; Yu *et al*., 2011). The employment of resistant cultivars has been the most efficient strategy to control this disease, but the enhancement has not been conducted successfully in crops thus far (Bhatnagar‐Mathur *et al*., 2015; Keneni *et al*., 2012; Reddy, 2016; Sunkara *et al*., 2014). A recent report has indicated that stable resistant varieties of peanut have been bred to overcome the incidence of serious bacterial wilt in large areas effectively. This report implies that peanut might contain resistant gene resources that are potentially important in controlling this disease. However, few resistant varieties of peanut have been developed in high yield and quality so far (Sunkara *et al*., 2014). Therefore, elucidating the molecular mechanism underlying the resistance of crops to bacterial wilt is urgently required to breed ideal varieties.

Plants have developed a complete defence mechanism against the infection of pathogens, such as bacteria, viruses, fungi and insects during evolution (Henry *et al*., 2013; Jones and Dangl, 2006; Thomma *et al*., 2011; Zvereva and Pooggin, 2012). Several pathogens are killed by the first defence system, whereas some are suppressed by the plant innate immune (PTI) system (Jones and Dangl, 2006; Zhang and Zhou, 2010). Notwithstanding, various successful pathogens deploy effectors for pathogen virulence. Many effectors can interfere with PTI to some extent as effector‐triggered susceptibility (Jones and Dangl, 2006). A given effector is ‘specifically recognized’ by plant NB‐LRR proteins (R genes) during effector‐triggered immunity (ETI) (Jones and Dangl, 2006). In general, R gene‐triggered resistance is associated with a rapid defence response termed hypersensitive response (HR) (Dangl *et al*., 1996; Greenberg, 1997; Keen, 1990; Thomma *et al*., 2011). HR brings a localized cell and tissue death at the infection site following a series of downstream defence responses (Baker *et al*., 1997; Lamb *et al*., 1989; Ryals *et al*., 1996; Zvereva and Pooggin, 2012).

NBS‐LRR genes are classified into two subfamilies, namely TIR‐NBS‐LRR and non‐TIR‐NBS‐LRR, on the basis of the motifs located in the N‐terminal region (Liu *et al*., 2007). The former subfamily contains a Drosophila Toll/mammalian interleukin‐1 receptor (TIR) domain, whereas the latter subfamily consists of a coiled coil (CC)/leucine zip motif (Van Ooijen *et al*., 2008). Thus far, more than 70 disease resistance genes have been cloned and characterized in monocots and dicots (Liu *et al*., 2007). Most of these genes are NBS‐LRR genes obtained using map‐based cloning and transposon tagging methods in crops (Hulbert *et al*., 2001; McDowell and Woffenden, 2003; Meyers *et al*., 2005; Takken and Joosten, 2000).

R gene products can directly or indirectly recognize pathogen effector proteins (avirulence protein) and induce resistance (Cesari *et al*., 2013; Flor, 1971; Sohn *et al*., 2014). Furthermore, some NB‐LRR proteins act downstream of R protein activation. The tobacco ‘N‐required gene 1’ and tomato ‘NB‐LRR protein required for HR‐associated cell death 1’ (NRC1) (both as CC‐NB‐LRR proteins) are required for TIR‐NB‐LRR protein N‐mediated resistance to tobacco mosaic virus and receptor‐like protein Cf‐4‐mediated resistance to tomato leaf mould, respectively (Gabriëls *et al*., 2007; Peart *et al*., 2005). The CC‐NB‐LRR activated disease resistance 1 family of proteins in *Arabidopsis* is required for salicylic acid (SA)‐dependent ETI (Bonardi *et al*., 2011). The downy mildew resistance locus RPP2 in *Arabidopsis* Col‐0 comprises two closely linked NB‐LRR genes, RPP2A and RPP2B, for resistance (Sinapidou *et al*., 2004). The rice *Pia* locus for blast (*Magnaporthe*) resistance includes two divergently transcribed CC‐NB‐LRR genes, RGA4 and RGA5, for resistance (Cesari *et al*., 2013).

Quantitative trait loci (QTL) controlling resistance to bacterial wilt have been identified in several crops, such as tomato (Carmeille *et al*., 2006; Danesh *et al*., 1994; Mangin *et al*., 1999; Thoquet *et al*., 1996; Wang *et al*., 2000), eggplant (Lebeau *et al*., 2013) and tobacco (Qian *et al*., 2012), as well as in model plants, such as *Arabidopsis thaliana* (Godiard *et al*., 2003) and *Medicago truncatula* (Ben *et al*., 2013). However, only two resistance genes have been identified thus far: the *A. thaliana ERECTA* gene involved in polygenic resistance and the *A. thaliana RRS1‐R* gene involved in monogenic resistance. *RRS1‐R* is a typical TIR‐NB‐LRR resistance gene generated through map‐based cloning in *Arabidopsis* (Deslandes *et al*., 2002). *RRS1‐R* contains a WRKY transcription factor domain at the C‐terminus to activate downstream gene expression and a nuclear localization signal (NLS) at its N‐terminus (Deslandes *et al*., 2002). PopP2 is the corresponding avirulence gene of *RRS1‐R*. It was recognized and recruited with the LRR domain of *RRS1‐R* and trafficked to the nucleus through NLS. ERECTA, a quantitative resistance locus for bacterial wilt, encodes a leucine‐rich repeat receptor‐like kinase. ERECTA‐controlled resistance is triggered by disease defence response through the phosphorylation of extracellular kinase‐regulated downstream genes (Godiard *et al*., 2003). However, resistance genes to bacterial wilt have yet to be cloned in crops other than *Arabidopsis*, thereby hindering genetic enhancement towards the disease. In addition, the molecular mechanism and details in the signalling pathway of R gene resistance to *R. solanacearum* have yet to be elucidated.

In this study, the up‐regulated NBS‐LRR resistant gene *AhRRS5* was screened from peanut through microarray analysis. This gene was induced by *R. solanacearum* containing the typically conserved motifs of an NBS‐LRR gene. AhRRS5 was localized in the nucleus and could be up‐regulated relatively higher in the resistant than susceptible peanut cultivars against bacterial wilt. This gene responded differently to phytohormones, such as salicylic acid (SA), abscisic acid (ABA), methyl jasmonate (JA) and ethephon (ET), among distinct resistance varieties. The transient overexpression of *AhRRS5* induced HR responses in *Nicotiana benthamiana*, whereas the overexpression of this gene in *Nicotiana tabacum* significantly enhanced the resistance of peanut to *R. solanacearum*. The underlying mechanism presumably involved the significant up‐regulation of several representative stress‐responsive and resistance marker genes. We concluded that *AhRRS5* indirectly participates in the defence response to *R. solanacearum* in plants through multiple signalling regulatory networks.

## Results

### Cloning and phylogenetic analysis of *AhRRS5*

**Figure 1:**
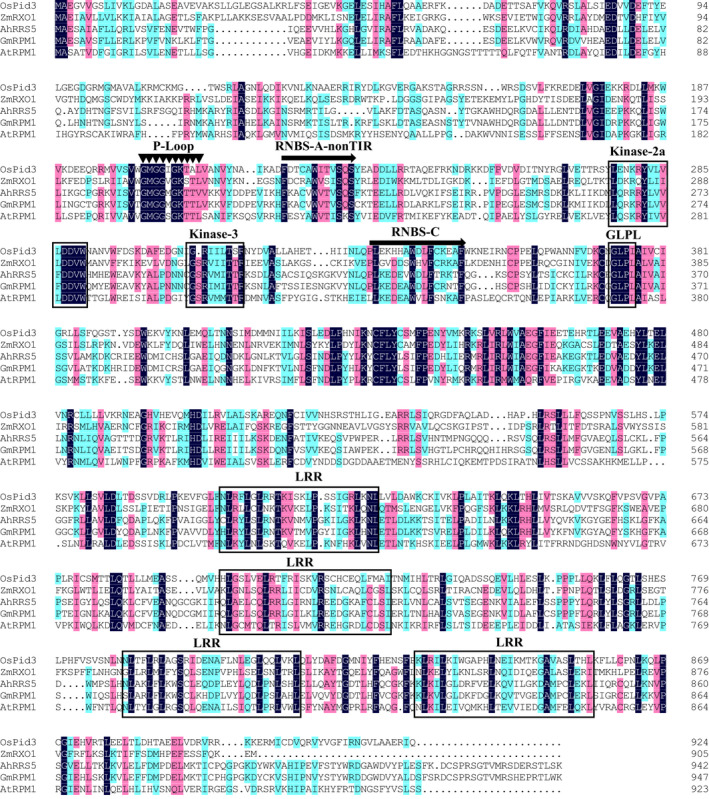
Conserved domain comparison between the deduced amino acid sequence of AhRRS5 and other resistance proteins. Sequences were aligned using the ClustalW2 program. Gaps have been introduced to optimize the alignment. Identical or conserved amino acids are shaded in dark and light, respectively. The sources of the proteins and GenBank accession numbers are as follows: OsPid3, blast resistance protein (ACN62383.1) from *Oryza sativa Indica Group*; AtRPM1 (AGC12570.1) from *Arabidopsis thaliana*; GmRPM1 (XP_006587620.1) from *Glycine max*; and ZmRXO1 disease resistance protein (AAX31149.1) from *Zea mays*.

The 5′ and 3′ unknown cDNA sequences of *AhRRS5* were cloned by rapid amplification of cDNA ends (RACE) on the basis of the known fragment. The full‐length cDNA sequence of *AhRRS5* was isolated from the total RNA of peanut leaf through reverse transcription polymerase chain reaction (RT‐PCR), and the genomic DNA sequence of *AhRRS5* was cloned from the genomic DNA of peanut through PCR. The full‐length cDNA contained a 3157‐bp open reading frame encoding a polypeptide of 943 amino acids, an 88‐bp 5′ untranslated terminal region (5′ UTR), and a 138‐bp 3′ UTR. The genomic DNA sequence of *AhRRS5* was 3662‐bp, including a 535‐bp intron. The entire sequence of the AhRRS5 protein has 76% identity with an NBS‐LRR resistance protein, RPM1‐like, in *Glycine max* (Figure 1; Data S1 and Data S2). A comparison of the AhRRS5 amino acid sequence with the R gene of a known function demonstrates that it most closely resembles RXO1 (33% identity and 53% positive) from *Zea mays*, which confers resistance to X. o. pv. *Oryzicola* containing *avrRxo1*, and *RPM1* (32% identity and 53% positive) from *A. thaliana*, resisting *Pseudomonas syringae pv. maculicola 1* containing AvrBand, AvrRpm1, and Pid3 (33% identity and 53% positive) from Rice and resisting *Magnaporthe oryzae* (Data S2). The former two were resistant to bacterial pathogens.

Sequence analysis showed that the deduced AhRRS5 protein contained conserved NBS motifs, such as P‐loop (MGGVGKT), GLPL (GLPLALK), kinase‐2 (LLVLDDVVW), kinase‐3a (GSRVLVTTR) and RNBS‐C (YEVxxLSDEEAWELFCKxAF) motif (Bertioli *et al*., 2003; Zheng *et al*., 2012), and 4 LRR‐conserved domains (LxxLxxLxxLxLxxC/A‐xx) (Leah McHale *et al*., 2006) (Figure 1; Data S1). On the basis of the conserved domains at the N‐terminus of the deduced NBS‐LRR genes, the *AhRRS5* gene had the typical structure of non‐TIR‐NBS‐LRR genes (Wan *et al*., 2012), with RNBS‐A‐non‐TIR (FnLxAWVCvSQxF) domains (Figure 1).

**Figure 2:**
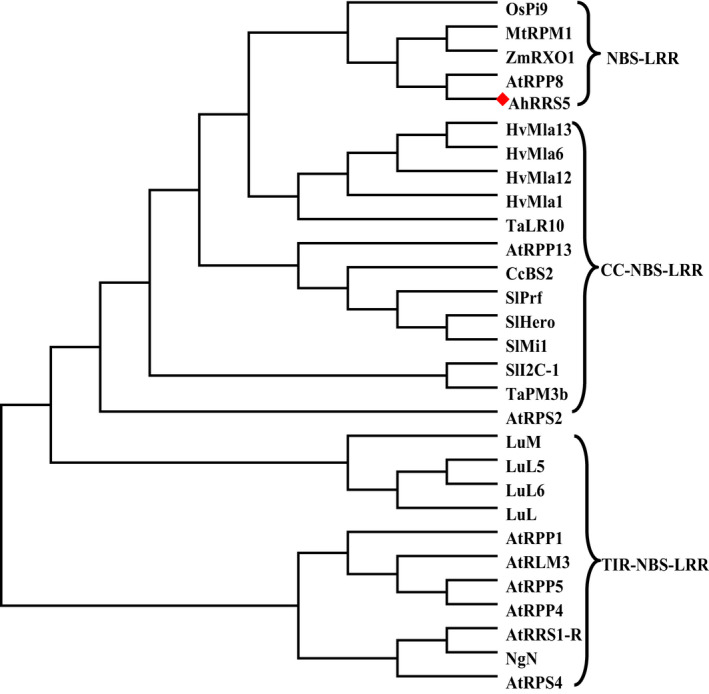
Phylogenetic tree was constructed using AhRRS5 and known different types of NBS‐LRR resistant proteins. AhRRS5 is shown by a red rhombus. Alignments were performed in ClustalW2, and phylogenetic tree was constructed by the neighbour‐joining algorithm of MEGA 5.01. Bootstrap values (1000 replicates) are shown in percentages at the branch nodes.

The phylogenetic analysis of 29 types of NBS‐LRR resistance proteins from GenBank together with AhRRS5 generated two clades coarsely (Figure 2; Data S3). The topology of the phylogenetic analysis showed that the NBS‐LRR‐type resistance proteins can be divided into two types, namely TIR‐NBS‐LRR and non‐TIR‐NBS‐LRR, and that the non‐TIR‐NBS‐LRR‐type resistance proteins can be subdivided into two classes, namely NBS‐LRR and CC‐NBS‐LRR. AhRRS5 is a NBS‐LRR‐type resistance protein that is similar to NBS‐LRR resistance proteins, such as RPM1 (XP_006587620.1|) from *Glycine max*, RPP8 (GenBank: XP_003612691.1) from *M. truncatula*, RXO1 (GenBank: AAX31149.1) from *Zea mays*, RPM1 (GenBank: AGC12590) from *A. thaliana* and Pi9 (GenBank: ABB88855.1) from *Oryza sativa*. These similarities indicate that these resistance genes share a common ancestor R gene and belong to NBS‐LRR‐type resistance genes (Figure 2).

### *AhRRS5* functions in the nucleus

**Figure 3:**
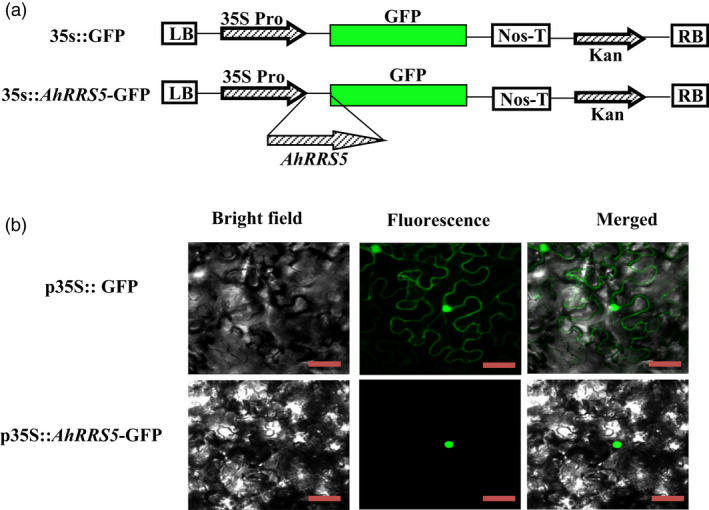
Subcellular localization of *AhRRS5*. (a) Schematic of p*35S::GFP* and p*35S:: AhRRS5‐GFP* constructs used for the subcellular localization of *AhRRS5* by agroinfiltration into *N. benthamiana* cells. (b) *AhRRS5*‐GFP localized in the nucleus of *N. benthamiana* cells, GFP alone localized throughout the whole cells. Bright field (left), fluorescence (middle) and merged images (right) were obtained at 48 h by using Leica confocal microscopy after agroinfiltration. Bars = 50 μm.

Sequence analysis indicated that the predicted AhRRS5 protein was localized in the nucleus (Data S1) (http://nls-mapper.iab.keio.ac.jp/cgi-bin/NLS_Mapper_form.cgi). To confirm this indication and the site of function, we generated an AhRRS5‐green fluorescent protein (GFP) fusion driven by the constitutive CaMV35S promoter (Figure 3a). With 35S::GFP as a negative control, the *AhRRS5::GFP* fusion gene was transformed into *Agrobacterium* strain GV3101, which was further infiltrated into *N. benthamiana* leaves. Typical results indicated the exclusive localization of AhRRS5‐GFP in the nucleus, whereas GFP alone occurred in multiple subcellular compartments, including the cytoplasm and the nucleus (Figure 3b). The results indicate that AhRRS5 is localized and functions in the nucleus.

### *AhRRS5* showed varied expression patterns among tissues

**Figure 4:**
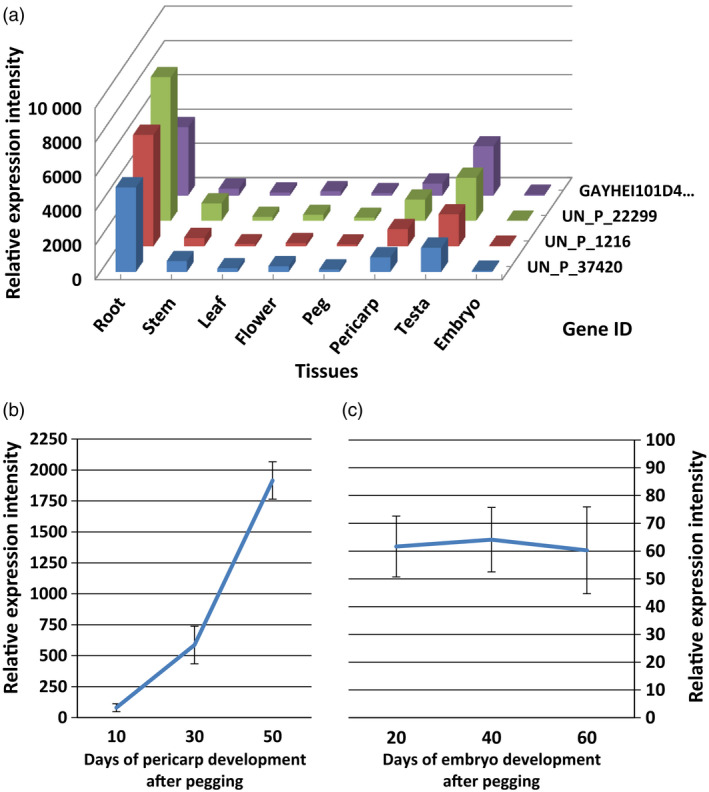
In silico identification of the expression characteristics of four members of the *AhRRS5* gene family. (a) The *AhRRS5* family showed tissue‐specific expression in peanut, the highest level was in the root, followed by the testa and pericarp. Weak expression was found in the other tissues. (b) *AhRRS5* genes increased expression with pericarp development. (c) AhRRS5 had the least expression levels with developing embryos. UN_P_37420, UN_P_1216, UN_P_22299 and GAYHEI101D4L7C_pchu_p are *AhRRS5* and the three other members of the same family.

In the microarray with a high density of unigenes, four unigenes including *AhRRS5* were found with a sequence identity of more than 97%. These unigenes apparently belong to the same *AhRRS5* gene family. Nonamplified double strain cDNA was used for microarray hybridization to evaluate the transcript levels of the unigenes. All four members showed a synchronized expression pattern among tissues or organs. They showed tissue‐specific expression manners; in particular, they were expressed the highest in the roots, then in the testa, pericarps and stem, but were weakly expressed in other tissues (Figure 4a). Embryos displayed the least expression levels of these genes. In addition, the transcripts of these genes obviously increased with pericarp development (Figure 4b) but remained almost constant with trace amounts during embryo development (Figure 4c). Therefore, *AhRRS5* may be involved in the resistant response and in plant development to some extent.

### *AhRRS5* showed a wide response to biotic and abiotic stresses

#### Response of *AhRRS5* to exogenous hormones

**Figure 5:**
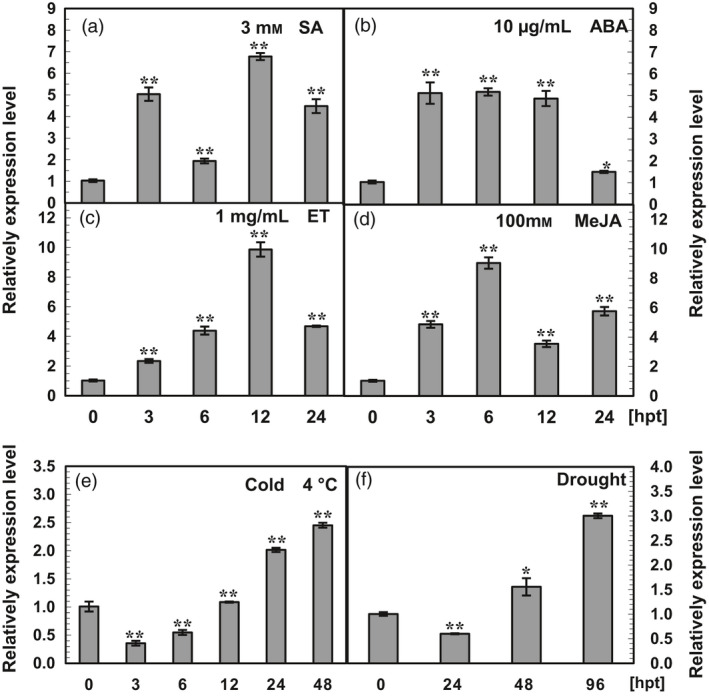
qRT‐PCR analysis of *AhRRS5* transcripts in peanut cultivar Minhua 6 under abiotic treatments. (a–d) *AhRRS5* relative expression level in peanut leaves at different time points after treatment with salicylic acid (SA, 3 mM), abscisic acid (ABA, 10 μg/mL), ethylene (ET, 1 mg/mL) and methyl jasmonate (MeJA, 100 mM). (e and f) *AhRRS5* expression was determined at various hour intervals after treatment with low temperature (4 °C) and drought in peanut plants at the eight‐leaf stage. The relative expression level of *AhRRS5* in peanut plants at various time points was compared with the mock control, which was set to 1. The asterisks indicate a significant difference (SNK test, *P < 0.05 or **P < 0.01). Error bars indicate the standard error.

The transcript level of *AhRRS5* was determined in the medium‐resistant variety Minhua 6 at the eight‐leaf stage after exogenous treatment with SA, ABA, ET and MeJA to identify the possible involvement of *AhRRS5* in signalling pathways relating to the phytohormones (Figure 5). Compared with the control plants, *AhRRS5* transcripts increased between 3 and 24 h with two peaks after SA treatment. The highest transcript level (6.7‐fold up‐regulation) was observed at 12 h post‐treatment (hpt) (Figure 5a). *AhRRS5* transcription also increased with a single peak of 5.1‐fold up‐regulation at 3 hpt after ABA treatment (Figure 5b). In response to ET, *AhRRS5* expression was enhanced from 3 hpt to 12 hpt, and the highest transcript level (10‐fold) was obtained at 12 hpt (Figure 5c). The application of 100 mM MeJA also elevated *AhRRS5* expression with two peaks, and the highest level was achieved at 6 hpt (Figure 5d).

**Figure 6:**
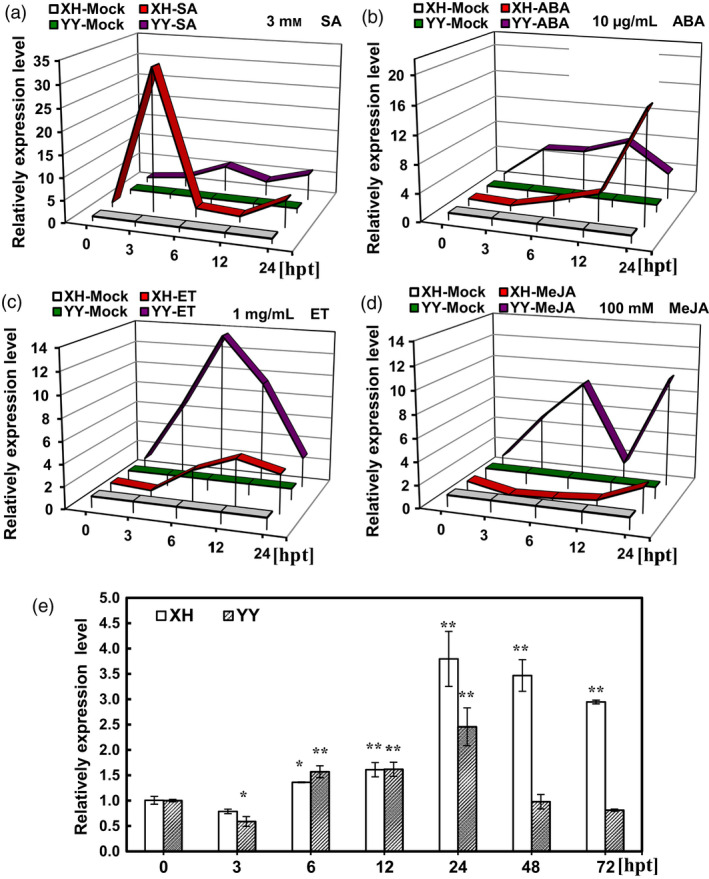
Comparative expression characteristics of *AhRRS5* between resistant and susceptible varieties under hormones and *R. solanacearum* treatments. (a) *AhRRS5* showed two expression peaks in response to SA within 24 h, and it up‐regulated over 32‐fold in susceptible variety at 3 HPT, much greater than in resistant one. (b) *AhRRS5* increased expression under ABA treatment with one peak; it up‐regulated later in the susceptible but >16‐fold at 24 h. (c) *AhRRS5* up‐regulated with one peak within 24 h with nearly 16‐fold at 6 h in the resistant variety. (d) *AhRRS5* responded differently between resistant and susceptible peanut with MeJA treatment, down‐regulated in the susceptible peanut and up‐regulated in the resistant ones with two peaks of over eightfold increase. (e) *AhRRS5* was up‐regulated higher in susceptible variety especially after 24 hpt with inoculation of *R. solanacearum*. XH‐Mock: susceptible variety Xinhuixiaoli without treatment; YY‐Mock: resistant variety Yueyou 92 without treatment. XH‐SA, XH‐ABA, XH‐ET and XH‐MeJA: susceptible variety treated with SA, ABA, ET and MeJA, respectively; YY‐SA, YY‐ABA, YY‐ET and YY‐MJA, resistant variety treated with SA, ABA, ET and MeJA, respectively. The relative expression level of *AhRRS5* in peanut plants at various time course was compared with mock or control, which was set to 1. The asterisk indicate a significant difference (SNK test,*P‐value <0.05 or **P‐value <0.01), Error bars indicate the standard error.

Highly susceptible and resistant varieties Xinhuixiaoli and Yueyou 92, respectively, were used to clarify the relationship between *AhRRS5* and the hormones (Figure 6). Although *AhRRS5* showed a similar expression in response to these hormones in Minhua 6, this gene demonstrated distinct expression characteristics between the two varieties. *AhRRS5* was more significantly up‐regulated after SA and ABA treatments in Xinhuixiaoli than in Yueyou 92 (Figure 6a,b); however, this gene increased less after ET and JA treatments (Figure 6c,d). In particular, the application of ET down‐regulated AhRRS5 in Xinhuixiaoli but up‐regulated it in Yueyou 92 (Figure 6d). This result indicates that the regulation of *AhRRS5* differs between resistant and susceptible varieties in peanut.

### Responses of *AhRRS5* transcripts to abiotic stresses

The responses of *AhRRS5* including three other orthologous NBS‐LRR genes to low temperature (4 °C) and drought were studied by microarray hybridization using the cDNA of mixed double strains at different time points (Materials and methods) in eight‐leaf Minhua 6. *AhRRS5* and three other NBS‐LRR genes remained constant in response to low temperature but were up‐regulated by nearly 8‐ to 10‐fold in response to drought (Data S4). To clarify whether *AhRRS5* is involved in the response to abiotic stresses, the relative transcripts of *AhRRS5* were also examined in eight‐leaf Minhua 6 seedlings under low temperature and drought treatments through quantitative real‐time PCR analysis (Figure 5e,f). The transcript level of *AhRRS5* decreased and then increased in response to low temperature and drought. In specific, under low temperature, the transcript level of *AhRRS5* decreased by two‐ to three‐fold at 3 and 6 hpt and then increased between 24 and 48 hpt, with the highest level (2.5‐fold) at 48 hpt (Figure 5e). Compared with the control, the transcript level of *AhRRS5* was down‐regulated by two‐fold at 1 day post‐treatment (dpt) but was up‐regulated from 2 dpt to 4 dpt with a 3.3‐fold induction at 4 dpt under drought (Figure 5f), thereby confirming the microarray results.

### Expression pattern of *AhRRS5* in the resistant/susceptible peanut cultivars after *R. solanacearum* challenge

*AhRRS5* was characterized using resistant and susceptible peanut cultivars after inoculation with *R. solanacearum* by microarray hybridization and qRT‐PCR. The four members of *AhRRS5* in the microarray exhibited similar pattern of transcription with *R. solanacearum* inoculation. These genes were up‐regulated by nearly one‐fold under inoculation with *R. solanacearum* in Yueyou 92, but a higher up‐regulation was observed in Xinhuixiaoli (Data S4). In addition, the expression patterns of *AhRRS5* at different time courses after *R. solanacearum* inoculation were compared in the two varieties. *AhRRS5* transcripts were induced between 0 and 24 h in the leaves of Yueyou 92 and then returned to their ground state at 72 hpi in response to *R. solanacearum* strain challenge. The expression level of *AhRRS5* in Xinhuixiaoli was up‐regulated from 6 hpi, showed a peak of 3.75‐fold transcript level at 24 hpi, and remained high between 24 and 96 hpi (Figure 6e). This finding suggests that *AhRRS5* participates in the immunity of peanut to *R. solanacearum*.

### Transient overexpression of *AhRRS5* in *N. benthamiana* leaves induces hypersensitive response

**Figure 7:**
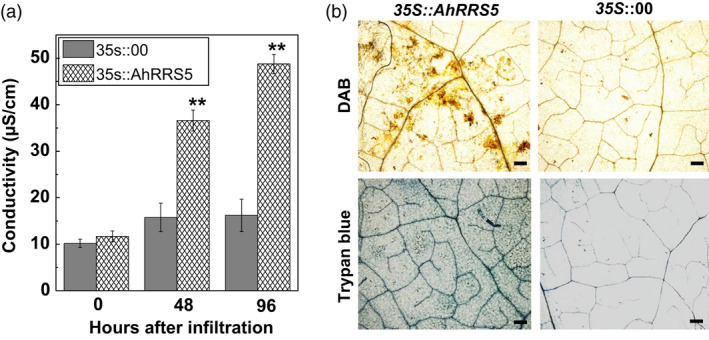
Effect of transient expression of *AhRRS5* in *Nicotiana benthamiana* on immunity induction. (a) Electrolyte leakage of *N. benthamiana* leaves were infiltrated with *Agrobacterium* strain GV3101 containing *35S::AhRRS5* and *35S::00*. (b) Trypan blue and DAB staining of cell death and H_2_O_2_ generation in *N. benthamiana* leaves 48 h after *AhRRS5*–*Agrobacterium* infiltration. Bars = 0.1 mm. Error bars indicate the standard error, Alphabet indicates statistically significant differences between wild‐type and *35S::AhRRS5* tobacco by Student–Newman–Keuls test, *P < 0.05 or **P < 0.01), Error bars indicate the standard error.

Successful pathogens can attenuate PTI by secreting effector molecules into the host plant cell. Some R proteins could recognize pathogen effector molecules and induce ETI with HR resulting in cell death at the infection site. This process is followed by a series of downstream defence responses. Overexpression vector harbouring p35S::AhRRS5 was generated and transformed into *Agrobacterium* GV3101 to verify whether AhRRS5 overexpression causes HR cell death. AhRRS5 was transiently expressed in *N. benthamiana* leaves through agroinfiltration. Then, AhRRS5 overexpression in *N. benthamiana* leaves induced an intensive HR mimicking cell death 48 h after infiltration. However, no visible HR cell death was found in those infiltrated with GV3101 harbouring empty vector p35S::00. Furthermore, electrolyte significantly leaked at 24 and 48 hpt after treatment, and darker trypan blue staining was observed after AhRRS5 overexpression for 24 hpt. This result suggests that AhRRS5 can trigger HR response in *N. benthamiana* leaves (Figure 7a,b). In addition, large amounts of H_2_O_2_ accumulation were found in the *N. benthamiana* leaves after AhRRS5 overexpression by DAB staining (Figure 7b). These results demonstrate that the transient overexpression of *AhRRS5* in tobacco leaves induces HR and H_2_O_2_ generation as a defence response to stresses.

### Overexpression of *AhRRS5* in tobacco enhances resistance to *R. solanacearum*

**Figure 8:**
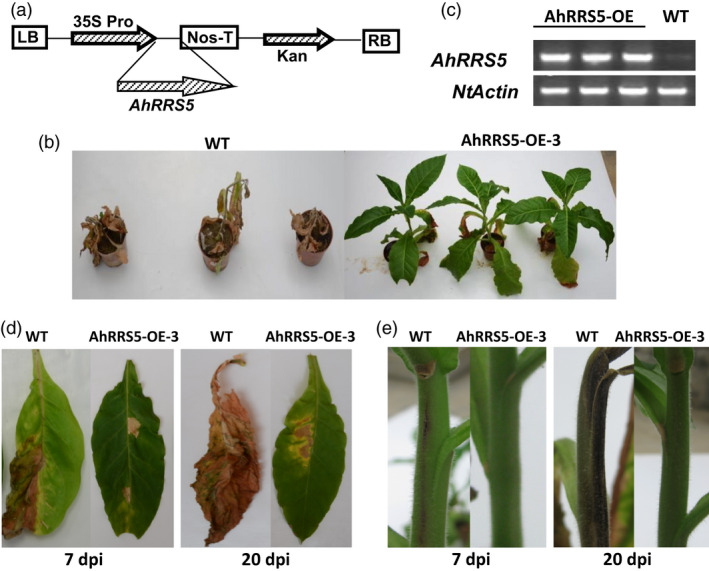
Overexpression of *AhRRS5* enhanced resistance to *Ralstonia solanacearum* in transgenic tobacco. (a) Schematic of the pBI121‐*AhRRS5* construct. LB and RB, left and right borders of the T‐DNA; 2 × 35SPro, two cauliflower mosaic virus 35S promoters; Nos‐T, NOS terminator; Kan^r^, kanamycin resistance. (b) The third leaves of 8‐week‐old wild‐type tobacco and AhRRS5‐OE‐3 transgenic plants were inoculated with 10 μL of suspension of 10^8^ cfu per millilitre of high‐virulence *R. solanacearum* strain. The photograph was obtained 20 days postinoculation (dpi). (c) RT‐PCR analysis of *AhRRS5* expression in transgenic and wild‐type tobacco plants; expression level of Ntactin was visualized as endogenous control. (d) Disease symptoms of detached leaves of wild‐type and AhRRS5‐OE‐3 transgenic plants after inoculation with *R. solanacearum*. Transgenic leaves showed immune resistance or high‐resistance phenotype. Photos were obtained at 7 and 20 dpi. (e) Different phenotypes of the stem were observed between wild‐type and transgenic AhRRS5‐OE‐3 plants after inoculation with *R. solanacearum*. Transgenic plant stem showed no or much week infections. Photos were taken at 7 and 20 dpi.

The involvement of *AhRRS5 R. solanacearum* resistance was evaluated by transforming CB‐1, a conventional tobacco cv., medium‐susceptible to bacterial wilt mediated by *Agrobacterium*, with *AhRRS5* driven by two copies of the CaMV35S promoter in the pBI121 binary vector. Transgenic T_0_ and T_1_ tobacco plants were generated to examine the role of *AhRRS5* in tobacco–*R. solanacearum* interaction (Figure 8a). Three T_2_ transgenic homozygous lines were screened by inoculation and identified for their resistance to *R. solanacearum* (Figure 8b). The line *AhRRS5‐OE‐3* line which showed the greatest *AhRRS5* relative transcript levels and resistant to *R. solanacearum* (not shown) of all the tested lines, was selected for the detailed disease resistance assays. No apparent phenotypic differences between the wild‐type and transgenic plants were observed. A highly virulent strain of *R. solanacearum* was used to inoculate individuals of *AhRRS5‐OE‐3* T_2_ lines and wild‐type plants. Vein injection was then used for *R. solanacearum* inoculation. All tested transgenic lines exhibited enhanced disease resistance. Evident wilting symptoms were detected in the leaves of wild‐type plants at 7 dpi, whereas only faint wilting symptoms were exhibited by *AhRRS5‐OE‐3* lines (Figure 8b,d). Extremely severe wilting symptoms were developed in the wild‐type plants at 20 dpi but not in the *AhRRS5‐OE‐3* transgenic lines. Wilting and contagion symptoms were evident on the stems of the infected wild‐type tobacco at 7 and 20 dpi, but no significant symptoms were found in the transgenic lines (Figure 8e). Further evaluation of *AhRRS5* was performed in the Honghuadajinyuan cultivar, which is hypersusceptible to *R. solanacearum*. Five transgenic T_2_ homozygous lines were inoculated compared with the wild type. All lines showed increased but distinct levels of resistance to *R. solanacearum* (Tables 1 and S2). Line 3 showed the highest resistance with a low infection index and a death rate of (7.08%) at 21 dpi, but the mock line showed serious wilting with 93.58% index and 81.08% death of plants, respectively, at 21 dpi. These results indicate that *AhRRS5* overexpression greatly enhances disease resistance against *R. solanacearum* in tobacco.

**Table 1:** Disease indexes and death ratios of different OE lines and the wild type after inoculation with *Ralstonia solanacearum*

OE lines	7 dpi		21 dpi	
	Disease index (%)	Death ratio (%)	Disease index (%)	Death ratio (%)
OE‐2	22.90	2.80	45.79	34.58
OE‐3	12.83	0.00	20.35	7.08
OE‐4	26.51	4.82	64.46	56.63
OE‐5	37.39	10.62	72.35	60.18
OE‐8	19.92	6.50	31.10	14.63
Wild type	73.65	22.97	93.58	81.08

dpi, days postinoculation.

To further confirm the role of *AhRRS5* in disease resistance and elucidate its possible molecular mode of action, transcriptional responses of known defence genes to overexpression of *AhRRS5* in noninoculated tobacco plants were investigated by qPCR (Data S5). We examined transcript levels of the HR‐associated genes *NtHIN1, NtHSR201*,* NtHSR203* and *NtHSR515* (Sohn *et al*., 2007), SA‐responsive genes *NtPR1a/c*,* NtPR3*,* NtPR4* and *NtNPR1* (Brogue *et al*., 1991; Ward *et al*., 1991), JA‐responsive *NtPR1b and NtPR2* (Sohn *et al*., 2007) and ET‐associated genes such as *NtEFE26* and *NtACS6* (Chen *et al*., 2003). Each of the tested tobacco genes was shown previously to be up‐regulated in response to pathogen infection (Chen *et al*., 2003; Rizhsky *et al*., 2002; Sohn *et al*., 2007). We found transcript levels of HR‐associated genes, such as *NtHIN1*,* NtHSR201* and *NtHSR515* to be increased by 3.3‐fold, 2.8‐fold and 3.3‐fold in the *AhRRS5‐OE‐3* line compared to wild‐type plants, respectively. Transcript levels of the SA‐responsive *NtPR1a/c*,* NtPR3*,* NtPR4* and *NtNPR1* genes were increased in *AhRRS5‐OE‐3* plants by 11.9‐fold, 3.0‐fold, 2.0‐fold and 3.0‐fold, respectively, while those of the JA‐responsive *NtPR2* and *NtPR1b* genes were 2.5‐fold and 4.0‐fold higher in *AhRRS5‐OE‐3* plants. These results show that *AhRRS5* overexpression enhances stress‐related gene expression compared to the wild‐type tobacco.

### Up‐regulation of marker genes in response to *R. solanacearum* infection

**Figure 9:**
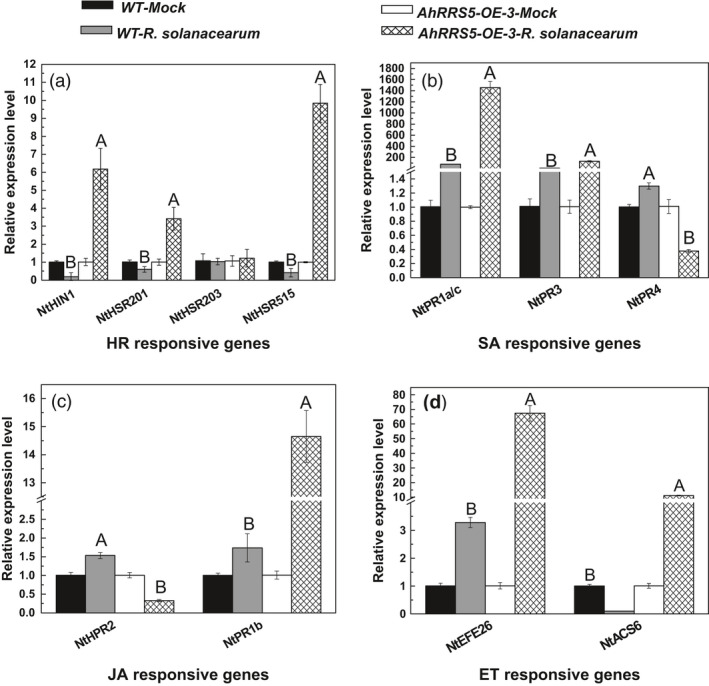
Transcript levels of tobacco defence‐related marker genes in wild‐type CB‐1 and *AhRRS5‐OE‐3* transgenic tobacco line 48 h after inoculation with *R. solanacearum*. The transcript levels of *NtHIN1*,* NtHSR201*,* NtHSR203*,* NtHSR515*,* NtPR1a/c*,* NtPR3*,* NtPR4*,* NtNPR1*,* NtPR2*,* NtPR1b*,* NtEFE26* and *NtACS6* were determined by quantitative real‐time PCR. Relative transcript levels were normalized using the transcripts of *NtEF1*α. The transcript levels of nontreated wild‐type or *AhRRS5‐OE‐3* tobacco plants were used as the control and assigned value of 1. Alphabet indicates statistically significant differences between wild‐type and *AhRRS5‐OE‐3* tobacco plants by Student–Newman–Keuls test (lowercase difference indicates *P* < 0.05; uppercase difference indicates *P* < 0.01).

HR‐responsive genes, namely *NtHIN1*,* NtHSR201* and *NtHSR515*, were significantly up‐regulated in the transgenic plants (*P* < 0.01 or *P* < 0.05) but down‐regulated in wild‐type CB‐1 to different extents at 48 hpi with *R. solanacearum* (Figure 9). By contrast, *NtHSR203* did not respond to the strain infection either in the transgenic or control plants (Figure 9a). The expression levels of *NtPR1a/c* and *NtPR3*, which are SA‐responsive pathogenesis‐related (PR) genes, increased in the *AhRRS5*‐OE‐1 plants by 1, 453.0‐ and 14.5‐fold, respectively, which are much higher than those in CB‐1. In addition, the *NtRP4* gene was down‐regulated by 2.5‐fold (Figure 9b). JA‐responsive *NtPR2* was up‐regulated in CB‐1 but down‐regulated in the transgenic plants in response to the strain, whereas *NtPR1b* was 14.3‐fold higher in the AhRRS5‐OE‐3 plants than in CB‐1 (Figure 9c). The transcript levels of ET‐responsive genes *NtEFE26* and *NtACS6* in the transgenic plants were also significantly increased at 48 h after infection but not in the wild‐type plants (Figure 9d). Several pathogen‐induced HR‐ and defence‐associated genes were enhanced by *AhRRS5* overexpression, but few were reduced or remained unchanged, which are consistent with the resistance enhancement in the transgenic lines. These findings indicate that *AhRRS5* functions in the resistance of transgenic tobacco through a wide series of signalling pathways.

### *NDR1* and *NPR1* genes were up‐regulated by *R. solanacearum* infection

**Figure 10:**
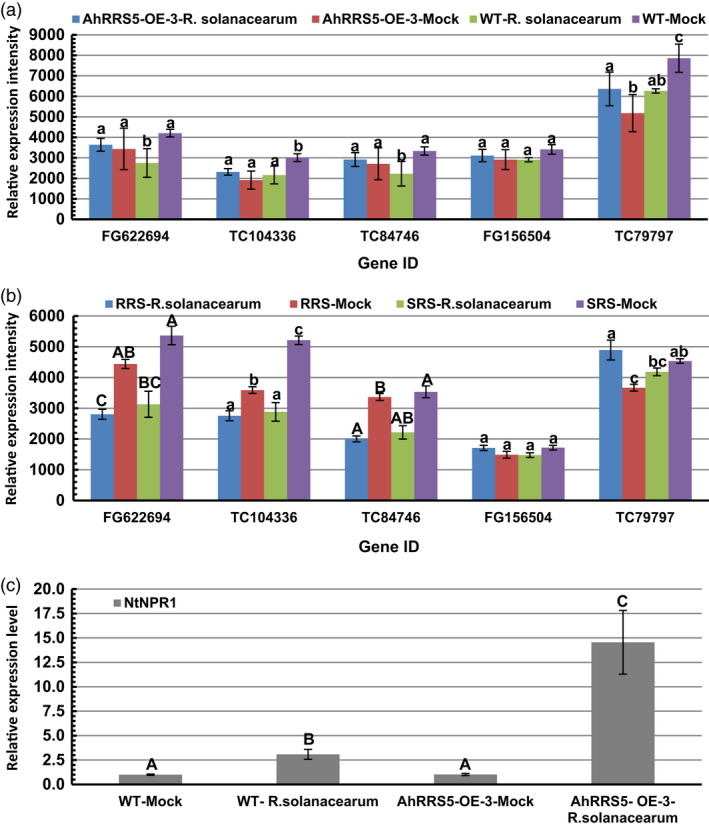
In silico and qPCR analysis of *NDR1*‐ and *NPR1*‐like gene expression upon inoculation with *R. solanacearum*. (a and b) Microarray data. (a) Expression of three *NDR1*‐like and two *NPR1*‐like genes. *AhRRS5‐OE‐3*‐*R. solanacearum* indicates tobacco CB‐1 cultivar transformed with *AhRRS5* with inoculation; *AhRRS5‐OE‐3*‐Mock, transgenic CB‐1 without inoculation; WT‐*R. solanacearum*, CB‐1 with inoculation; WT‐Mock, CB‐1 without inoculation. (b) Down‐regulation of three *NDR1*‐like genes in varieties after inoculation. RRS *‐R. solanacearum* indicates hyper‐resistant tobacco variety Yanyan 97 under inoculation; RRS‐Mock, hyper‐resistant variety Yanyan 97 without inoculation. SRS *R. solanacearum*, hypersusceptible variety Honghuadajinyuan with inoculation; SRS‐Mock, hypersusceptible variety Honghuadajinyuan without inoculation. *FG622694*, *TC104336* and *TC84746* are *NDR1*‐like genes; *FG156504* and *TC79797* are *NPR1/NIM1*‐like genes, respectively. (c) Transcript level of *NtNPR1* gene in tobacco plants with or without inoculation with *R. solanacearum t*hrough qRT‐PCR analysis. WT‐Mock and WT‐*R. solanacearum*,* AhRRS5‐OE‐3‐*Mock and *AhRRS5‐OE‐3*‐*R. solanacearum* indicate wild‐type tobacco without or with inoculation with pathogen, AhRRS5‐OE‐3 transgenic tobacco without or with inoculation with pathogen, respectively. Alphabets mark statistically significant differences between wild‐type and transgenic tobacco plants, by Student–Newman–Keuls test (lowercase differences indicate *P*‐value <0.05; uppercase differences indicate *P*‐value <0.01).

Non‐race‐specific disease resistance 1 (*NDR1*) and nonexpressor of pathogenesis‐related gene 1 (*NPR1*) genes were involved in the R gene resistance signalling pathway. *In silico* identification of three *NDR1*‐like and two *NPR1*‐like gene expressions were performed between *AhRRS5‐OE‐3* transgenic plants and wild‐type plants, as well as hyper‐resistant and hypersusceptible varieties Yanyan 97 and Honghuadajinyuan after inoculation with *R. solanacearum*, respectively (Figure 10). Two *NPR1*‐like genes were slightly up‐regulated by 6%–23% in the *AhRRS5‐OE‐3* lines after inoculation but were down‐regulated by 15%–21% in the wild‐type plants after inoculation (Figure 10a). The *NPR1* gene, TC79797, considerably increased or decreased in response to the pathogen, consistent with the resistant and susceptible varieties after inoculation (Figure 10b). Furthermore, the results of real‐time PCR revealed that the transcript level of *NPR1* increased by 14.5‐fold in transgenic lines of *AhRRS5‐OE‐3* as compared with wild‐type plants after inoculation with *R. solanacearum,* much higher than the increase of transcripts in inoculated wild‐type over corresponding mock plants (Figure 10c).

The transcript levels of three *NDR1*‐like genes slightly increased in the *AhRRS5* transgenic lines but significantly decreased in the wild type after inoculation; this result indicates that *AhRRS5* can maintain a high level of expression for the *NDR1* gene (Figure 10a). However, three *NDR1‐*like genes were considerably down‐regulated in both resistant and susceptible varieties after inoculation (Figure 10b). The results indicate that both *NDR1*‐ and *NPR1*‐like genes in tobacco are involved in *AhRRS5* resistance in transgenic tobacco, but only *NPR1* genes are required for the hyper‐resistant tobacco variety Yanyan 97. *AhRRS5* might also be involved in the R gene signalling for resistance against microbial infection.

## Discussion

### *AhRRS5* is a novel peanut NBS‐LRR resistance protein localized in the nucleus

NBS‐LRR genes are a class of resistance genes that function in pathogen recognition and defence response signal transduction (Ameline‐Torregrosa *et al*., 2008; Gao *et al*., 2010). More than 70 disease resistance genes cloned from higher plants by map‐based methods belong to NBS‐LRR domain genes resistant to bacterial, fungal and viral diseases, as well as some environmental stresses (Liu *et al*., 2007). *AhRRS5* was isolated from peanut using microarray analysis and could be up‐regulated by *R. solanacearum* inoculation. The AhRRS5 protein has a typical NB‐ARC domain containing P‐loop, kinase‐2, kinase‐3a and GLPL and other conservative modules similar to *Arabidopsis RPM1*, RXO1 protein of maize, Pid3 of rice and so on (Figure 1; Leister *et al*.,^1996^; Zhao *et al*., 2004; Chen *et al*., 2011). Four normal LRR motifs, which may participate in the peanut pathogen interaction or defence responses against the pathogen, were found in AhRRS5 (Takken and Joosten, 2000). The revealed amino acid sequence of AhRRS5 most closely resembles those of R genes of known functions, such as RXO1 from *Z. mays* resistant to *Xanthomonas oryzae* pv. *Oryzicola* (Zhao *et al*., 2004, 2005), RPM1 from *A. thaliana* resistant to *P. syringae* (Leister *et al*., 1996) and Pid3 from rice resistant to *M. oryzae* (Chen *et al*., 2011) (Data S2). Phylogenetic analysis with 29 R genes of known functions showed that AhRRS5 could be classified into non‐TIR‐NBS‐LRR type and NBS‐LRR subclass of resistance genes.

Subcellular localization visualized by the AhRRS5::GFP fusion protein in *N. benthamiana* leave cells showed that the AhRRS5::GFP fusion protein appeared solely in the nucleus and was associated with its nuclear localization signal GKFKKLKILGLDRF at positions 816–829 (Figure 3b; Data S1). This result agrees with the subcellular localization features of most NBS‐LRR disease resistance genes (Meyers *et al*., 2003). The first identified resistance gene to bacterial wilt is *RRS1‐R* (a TIR‐NBS‐LRR gene) in *Arabidopsis*, which is mainly cytoplasm‐localized but nuclear‐localized only depending on the presence of effector PopP2 from *R. solanacearum* (Deslandes *et al*., 2003). The RRS1‐R protein contains TIR‐, NBS‐ and LRR‐conserved domains, aside from a WRKY motif, which activates transcription in plants (Eulgem and Somssich, 2007). Another NBS‐LRR resistance gene, *RPS4*, to *R. solanacearum* in *Arabidopsis* is also localized in both the nucleus and cytoplasm (Wirthmueller *et al*., 2007). However, RPM1 activated in the plasma membrane functions independent of the nucleus (Boyes *et al*., 1998; Gao *et al*., 2011). Therefore, AhRRS5 possibly functions mainly in the nucleus.

### *AhRRS5* is widely involved in defence responses to biotic/abiotic stresses

*AhRRS5* transcripts were up‐regulated in both resistant and susceptible varieties challenged with *R. solanacearum* and highly up‐regulated in the susceptible variety at 24 hpi (Figure 6e). These results indicate that *AhRRS5* participates in the defence response to the pathogen. *AhRRS5* was up‐regulated in response to all exogenous hormones applied, namely SA, ABA, ET and JA, in the leaves, although this gene was specifically expressed in the peanut root, testa and pericarp, and weakly in other organs, such as the leaf (Figure 4a). These phytohormones are well‐known signalling molecules involved in controlling the defence gene expression against biotic and abiotic stresses (Divi *et al*., 2010; Ton *et al*., 2009). SA is usually associated with R gene‐mediated disease resistance, and SA‐deficient mutants often compromise R gene‐mediated resistance (Yang *et al*., 2013). Exogenous application of SA induces PR genes and enhances resistance to a broad range of pathogens (Bari and Jones, 2009). *Arabidopsis* RRS1‐R‐mediated resistance to *R. solanacearum* is partially dependent on SA and NDR1 (Deslandes *et al*., 2002). *Arabidopsis* RCY1 gene, which encodes a CC‐NBS‐LRR protein for resistance to the yellow strain of cucumber mosaic virus, requires SA and ET signalling (Takahashi *et al*., 2002). ET regulates various growth and developmental processes and is also involved in responses to stresses, such as salt, drought, cold, flooding and infection caused by microbes and insects (Yoo *et al*., 2009). ET could modulate disease resistance (Broekaert *et al*., 2006; Van Loon *et al*., 2006). MeJA regulate defence to herbivores and necrotrophic pathogens (Browse, 2009). SA and JA/ET defence pathways are usually antagonistic, but synergistic interactions have also been reported in defence response to pathogens (Beckers and Spoel, 2006; Mur *et al*., 2006; Nahar *et al*., 2012; Vos *et al*., 2015), which is also consistent with the results on *AhRRS5* responding to phytohormones such as SA, JA and ET. Rice ET, JA and SA biosynthetic pathways are prerequisites for defence against *Hirschmanniella oryzae*, and ABA participates in the antagonistic interaction to SA/JA/ET‐dependent basal defence to the pathogen (Nahar *et al*., 2012). We found *AhRRS5* was up‐regulated in response to all of the four hormones including ABA. ABA functions in abiotic stress tolerance, antagonizes the SA signalling pathway in higher plants and enhances disease susceptibility (Bari and Jones, 2009; Jiang *et al*., 2010; Nahar *et al*., 2012). However, ABA plays a positive role in papilla‐mediated defence against *Leptosphaeria maculans* in *Arabidopsis* (Ton *et al*., 2009). Exogenous application of ABA strengthens rice basal resistance against the brown spot caused by *Cochliobolus miyabeanus* (De Vleesschauwer *et al*., 2012). The role of ABA in defence depends on the type of pathogens, timing of the defence response and plant tissues (Ton *et al*., 2009). In general, hormone balance plays a vital role in fine tuning appropriate defence responses to the recognized pathogen.

In the present study, the results of qRT‐PCR and microarray analysis showed that *AhRRS5* was up‐regulated by SA, ABA, ET and JA and was enhanced differently in the response to *R. solanacearum* in three resistant varieties. Concentration curves showed that *AhRRS5* was up‐regulated with two optimal peaks in response to SA and JA, but with a single peak to ABA and ET in Minhua 6 (Figure 5a–d). Similar patterns were also found in Xinhuixiaoli and Yueyou 92, although *AhRRS5* was down‐regulated in Xinhuixiaoli 24 h after JA treatment (Figure 6a–d). These results indicate that *AhRRS5* may involve in the crosstalk between these phytohormones against pathogen infection, such as *R. solanacearum*. *AhRRS5* also showed an altered response to low temperature and drought (Figure 5e,f), indicating its association with biotic/abiotic stresses. Our data suggest that peanut AhRRS5 plays a role in the defence response to bacterial wilt via the synergistic interaction of diverse signalling pathways. Therefore, *AhRRS5* in response to *R. solanacearum* may adopt a distant mechanism in comparison with other pathogen‐associated genes.

### *AhRRS5* confers resistance to bacterial wilt in heterozygous tobacco transformant

The resistance genes against *R. solanacearum* have not been cloned and characterized except for model plant *Arabidopsis* (Deslandes *et al*., 2002; Godiard *et al*., 2003). *AtRRS1‐R*, genetically identified as recessive, confers dominant resistance to *R. solanacearum* GMI1000 in transgenic *Arabidopsis*. This gene presents a novel R gene structure combining domains of a TIR‐NBS‐LRR protein and a WRKY motif (Deslandes *et al*., 2002). Deslandes *et al*. (2003) showed that RRS1 can recognize the pathogen by directly interacting with effector PopP2 and depends on PopP2 to colocalize at the nucleus for pathogen defence. The *Arabidopsis* LRR‐RLK gene ERECTA, located in the QTL QRS1, shows resistance to *R. solanacearum* and also affects the development of aerial organs (Godiard *et al*., 2003). The NB‐LRR gene RPS4 from *Arabidopsis* ecotype Ws‐0 functions as a dual resistance gene system with RRS1 to prevent three distinct pathogens, namely *R. solanacearum*,* Pst‐avrRps4* and *Colletotrichum higginsianum* (Narusaka *et al*., 2009). *RPS4* was suggested to function downstream of, or together with, *RRS1‐Ws* in the signalling pathway resistant to *R. solanacearum*.

*AhRRS5* induced by *R. solanacearum* challenge is a non‐TIR‐NBS‐LRR gene different from *RRS1‐R* in *Arabidopsis*. Overexpression transgenic tobacco constitutively expressing *AhRRS5* showed enhanced disease resistance to bacterial wilt. In specific, *AhRRS5* overexpression in transgenic CB‐1, a medium‐susceptible cultivar, showed strong resistance to the pathogen infection (Figure 8). The hypersusceptible cultivar Honghuadajinyuan overexpressing *AhRRS5* also increased the resistance to *R. solanacearum* infection, although different transgenic lines demonstrated distinct levels of resistance in response to the pathogen (Table 1). Lines OE‐3 and OE‐8 showed much higher resistance or immune response to bacterial wilt than other lines, which may have resulted from the effect of insertion locations of the gene in chromosomes. The transient overexpression of *AhRRS5* in *N. benthamiana* showed that it can induce hypersensitive response causing cell death and also produce H_2_O_2_ in HR (Figure 7). These results indicate that *AhRRS5* may participate in resistance against *R. solanacearum* involving ROS signalling. Therefore, *AhRRS5* is a novel NBS‐LRR resistance gene cloned from peanut, which confers resistance to the *R. solanacearum*.

### AhRRS5 resistance is involved in multidefence signalling pathways

A complex network of different signalling transductions exists in plant–pathogen interactions, and different signalling pathways are associated with the transcription of some marker genes in their mediated disease resistance reaction. Many marker genes, such as *NtHIN1*,* HSR201* and *HSR515*, are activated in HR signalling (Sohn *et al*., 2007). SA‐mediated defence responses could activate system‐acquired resistance (SAR) and are accompanied with the expression of several PR genes, such as *PR1a/c*,* PR3*,* PR4* and *PR5* (Dong, 1998; Glazebrook, 2005). PR genes *PR2* and *PR1b* are activated and expressed in ET‐mediated defence response, whereas EFE26 and ACS6 are activated in JA‐mediated defence response (Koornneef and Pieterse, 2008; Kunkel and Brooks, 2002; Thomma *et al*., 1998). Changes in the expression levels of these markers directly indicate the involvement of plant defence responses and signal transduction pathways (Chen *et al*., 2003; Rizhsky *et al*., 2002; Sohn *et al*., 2007). We examined the transcripts of these marker genes in *AhRRS5* overexpression tobacco lines by qPCR. Results showed that *AhRRS5* overexpression up‐regulated not only the transcript levels of *NtHIN1*,* NtHSR201* and *NtHSR515* in HR signalling but also those of SA‐ regulated genes (*PR1a/c*,* PR3*) in the T_2_ tobacco plants inoculated with virulent *R. solanacearum* (Figure 9a,b). The transcript levels of JA‐regulated *PR1b* and ET‐responsive *NtEFE26* and *NtACS6* were also greatly enhanced (Figure 9c,d). The results conform to the data in peanut, in which *AhRRS5* was up‐regulated by the exogenous applications of SA, ET, JA and ABA. The RRS1‐R‐mediated bacterial wilt resistance in *Arabidopsis* involves ABA participation, and the effect of ABA is greater than that of SA (Deslandes *et al*., 2003; Hernández‐Blanco *et al*., 2007). These results are relatively similar to *AhRRS5* response to *R. solanacearum*, indicating that these hormone signals perform synergistically against the pathogen. The overexpression of *AhRRS5* conferring increased resistance to bacterial wilt in tobacco was achieved by the increase the gene expression in defence signal transduction pathways.

### *AhRRS5* resistance requires the involvement of *NDR1* and *NPR1*

*AhRRS5* overexpression up‐regulated *NDR1* transcripts in response to *R. solanacearum* challenge, concurring with the report that *RRS1‐R* in *Arabidopsis* is SA‐dependent and requires the downstream gene *NDR1* for its resistance to bacterial wilt (cf., Chen *et al*., 2003). However, *NDR1* was significantly down‐regulated in the nontransgenic resistance variety Yanyan 97 in response to the pathogen. This finding indicates that other resistance mechanisms exist in response to bacterial wilt. *NDR1* primarily mediates signalling derived from the CC‐NB‐LRR type of R proteins, whereas *EDS1* involves those from the TIR‐NB‐LRR class of R proteins (Aarts *et al*., 1998; Wang *et al*., 2014). These results are apparently contradictory to the events of *AhRRS5* and *AtRRS1‐R* (Deslandes *et al*., 2002; Lahaye, 2002). *NDR1* involves R protein‐mediated resistance to many pathogens (Day *et al*., 2006; Lu *et al*., 2013; Repetti *et al*., 2004). Soya bean *GmNDR1a* and *GmNDR1b* bind pathogen effectors and regulate resistance signalling (Selote *et al*., 2014). *Arabidopsis* resistance signalling pathways to *P. syringae* 2 and *P. syringae* pv. *maculicola 1* exhibit different mechanisms of activation in terms of effector action, but both require NDR1 participation (Kim, 2006). Thus, *AhRRS5* is associated with NDR1 for its mediated resistance to bacterial wilt.

*NPR1* is a key regulator of SAR and is essential for the SA signal transduction to activate PR gene expression (Pieterse and Van Loon, 2004; Sandhu *et al*., 2009). We examined *NPR1* transcription by employing microarray analysis and found that the transgenic plants overexpressing *AhRRS5* up‐regulated the two *NPR1* transcripts after inoculation with *R. solanacearum* but down‐regulated them after pathogen challenge in wild‐type plants (Figure 10a,b). These results were confirmed in the resistant and susceptible varieties, indicating that *NPR1* plays an important role in pathogen resistance. We further found that the *AhRRS5‐OE‐3* line significantly up‐regulated the transcript level of *NPR1* by 14.5‐fold in response to the *R. solanacearum* challenge (Figure 10c). The PR marker genes of SA signalling in the transgenic plants of *AhRRS5* were then up‐regulated (Figure 9b). *NPR1*‐mediated signalling resisting viral and bacterial pathogens and repressing *NPR1* transcript would increase the susceptibility of plants to pathogens (Li *et al*., 2012; Xiao and Chye, 2011). Thus, our results suggest that *AhRRS5* participates in pathogen resistance by employing the *NPR1*‐mediated SA signalling and the R gene pathway associated with *NDR1*.

## Experimental procedures

### Plant materials and growth conditions

Peanut cultivars (*Arachis hypogaea* cv. Minhua 6, cv. Yueyou 92 and cv. Xinhuixiaoli, as medium‐resistant, hyper‐resistant and hyper‐susceptible variants to *R. solanacearum*, respectively) were provided by the Oil Crop Institute in Fujian Agriculture and Forestry University. Seeds were sown in sterile sands in plastic pots. Seedlings of transgenic lines and wild‐type tobacco (*Nicotiana tabacum* cv. CB‐1, cv. Yanyan 97 and cv. Honghuadajinyuan, with medium susceptibility, hyper‐resistance and hyper‐susceptibility to *R. solanacearum*, respectively) were provided by Fujian Tobacco Agricultural Research Institute. *N. benthamiana* is available in this laboratory. T_2_ seeds of transgenic tobacco lines were surface‐sterilized with 75% alcohol for 20 sec, 10% H_2_O_2_ for 10 min, washed five times with sterile water and finally placed on MS medium supplemented with 75 mg/L kanamycin for 2–3 weeks. The survivals were then transferred into a soil mix containing peat moss/perlite (2/1, v/v) in a plastic tray and grown in a greenhouse for another 2–3 weeks. Transgenic and wild‐type tobacco plants of the same size were transferred into a soil mix containing peat moss/general soil (2/1, v/v) in plastic pots for another 3–4 weeks. Peanut and tobacco plants were grown in the greenhouse at 26 °C and 70% relative humidity under a 16 h/8 h light/dark cycle.

### Pathogens and inoculation

Virulent strains *Rs‐P.362200* and FJ1003 strain of *R. solanacearum* were from peanut and tobacco, respectively. The pathogen strains were streaked on TTC agar medium (0.5 g/L 2,3,5‐triphenyltetrazolium chloride, 5 g/L peptone, 0.1 g/L casein hydrolysate, 2 g/L D‐glucose and 15 g/L agar) (Kelman, 1954) and then incubated at 28 °C for 48 h. Virulent colonies were harvested with sterile water (with 0.02% Tween‐20), and the inoculum was prepared by adjusting the concentration of bacterial cells to an optical density of 0.5 at 600 nm wavelength (NanoDrop 2000c; Thermo Fisher Scientific, San Jose, CA, USA), corresponding to approximately 10^8 ^cfu/mL.

Then, 4‐week‐old peanut seedlings of Yueyou 92 and Xinhuixiaoli were inoculated at the third and fourth leaves from the upperpart by leaflet cutting (perpendicular to the midrib of leaflet, 2/3 deep cut to the midrib), and four leaflets were inoculated per plant. Control plants were inoculated with distilled water containing 0.02% Tween‐20. Two uncut leaflets of the treated leaves were harvested at the indicated time points for future analysis.

Tobacco was inoculated by infiltrating 10 μL of *R. solanacearum* suspension with 10^8 ^cfu/mL concentration into the third leaves from the upperpart using a syringe with a needle, and then, the fourth leaves were harvested at the indicated time points for future analysis. The typical symptoms of bacterial wilt were monitored daily in five disease severity ratings from 0 to 4, where 0 = no symptoms, 1 = 1/4 inoculated leaves wilted, 2 = 1/4−1/2 inoculated leaves wilted, 3 = 1/2−3/4 inoculated leaves wilted and 4 = whole plant wilted, plant death. Disease index (DI) and death ratio (DR) were calculated using the following formula: DI (%) = [∑ (ni × vi) ÷ (*V* × *N*)] × 100, DR (%) = (ni ÷ *N*) × 100, where ni = number of plants with the respective disease rating; vi = the disease rating; *V* = the highest disease rating; and *N* = the total number of observed plants.

### Application of plant hormones and abiotic/biotic stresses

One‐month‐old peanut seedlings (Minhua 6) were sprayed with 3 mM SA, 10 μg/mL ABA, 1 mg/mL ET and 100 mM MeJA in distilled water (H_2_O). Control seedlings were sprayed with distilled water (H_2_O). The leaves of the treated seedlings were harvested at indicated time points, frozen in liquid nitrogen and then stored at −80 °C until used. Yueyou 92 and Xinhuixiaoli were used in another trial. Seven‐leaf peanut Minhua 6 plants were treated at 4 °C and 25 °C. Leaves were harvested at indicated time points after treatments. Minhua 6 plants at the seven‐leaf stage were treated by stopping and normal watering for drought stress. Leaves were harvested at different time points, frozen in liquid nitrogen and then stored at −80 °C until use. Three biological replicates were set for all stress treatments.

### Full‐length cDNA cloning

The candidate gene was screened through microarray analysis with approximately 100 000 unigene probes on the basis of the available fragment sequence. The 5′‐ and 3′‐end cDNA sequences were cloned by RACE using the SMART™ RACE cloning kit (Clontech, Palo Alto, CA) in accordance with the manufacturer's instructions with minor revisions. Total RNA was extracted from the leaves of resistant peanut cultivar to *R. solanacearum* by the CTAB method. RACE‐F and 3′ PCR adaptor primers were joined on both ends of the cDNA. Then, 5′ RACE was generated by PCR using the primary primer set of RACE‐F primer and PRRS_1EW9‐R, followed by the reaction system: 94 °C for 5 min; 35 cycles of 30 s at 95 °C, 30 s at 60 °C and 1 min 30 s at 72 °C; and 72 °C for 10 min. Similarly, 3′ RACE was generated by the set of PRRS_1EW9_F and the 3′ PCR primer with the following PCR programme: 94 °C for 5 min; 5 cycles of 30 s at 95 °C and 2 min at 72 °C; and 30 cycles of 30 s at 95 °C, 60 °C, 30 s, 2 min at 72 °C; and 72 °C for 10 min. The RACE products were cloned and sequenced. After assembly, full‐length cDNA and DNA sequences of *AhRRS5* were cloned from the reverse transcription products and genomic DNA by using the set of AhRRS5‐ FL‐F and AhRRS5‐FL‐R. All primers used in this study are listed in Table S1.

### Sequence analysis and phylogenetic tree construction

*AhRRS5* sequence similarity analysis was performed using BLASTN and BLASTX (http://www.ncbi.nlm.nih.gov/BLAST). Four known functional resistant proteins with close similarities were obtained from the BLASTX results. Multiple sequence alignments were performed with ClustalW2 (Data S1). A phylogenetic tree was generated using 29 resistant proteins of known function by using MEGA 5.10 (Data S3).

### Subcellular localization

The full‐length *AhRRS5* ORF without the termination codon was amplified by high‐fidelity PCR polymerase with gene‐specific primers AhRRS5‐BamH1‐F and AhRRS5‐Asc1‐R harbouring BamHI and AscI sites, respectively. The PCR products were inserted into the vector pBI‐GFP between *BamHI* and *AscI* and formed a construct with the p35S::AhRRS5‐GFP fusion gene. With pBI‐GFP containing 35S::GFP as a control, p35S::AhRRS5‐GFP and p35S::GFP were transformed into *Agrobacterium tumefaciens* strain GV3101. which was cultured in induction medium (10 mM ethanesulfonic acid, pH 5.7, 10 mM MgCl_2_ and 200 mM acetosyringone), harvested and diluted to OD_600_ = 0.8, and then injected into *Nicotiana benthamiana* leaves using a syringe without a needle. Forty‐eight hours after agroinfiltration, GFP fluorescence was imaged in a fluorescence microscope, with an excitation wavelength of 488 nm and a 505–530 nm bandpass emission filter. GFP florescence was imaged using laser confocal florescence microscopy (Leica TCS SP8, Solms, Germany).

### Vector construction and transient expression

The complete ORF of *AhRRS5* was amplified by high‐fidelity PCR polymerase with AhRRS5‐OE‐F and AhRRS5‐OE‐R primers harbouring *BamHI* and *AscI* sites, respectively. The PCR products were cloned into the modified vector pBI121‐GUSA between *BamHI* and *AscI* sites to replace the GUSA gene. The obtained vector containing *AhRRS5* driven by the 2 × CaMV35S promoter was named *p35S::AhRRS5*. The *p35S::AhRRS5* vector was transferred into *Agrobacterium tumefaciens* strains GV3101 and EHA105.

*Agrobacterium tumefaciens* strain GV3101 harbouring the *p35S::AhRRS5* vector was cultured to OD_600_ = 1.0 in induction medium (10 mM ethanesulfonic acid, pH 5.7, 10 mM MgCl_2_ and 200 mM acetosyringone) and diluted to OD600 = 0.8. Th diluted culture was injected into *Nicotiana benthamiana* leaves using a syringe without a needle. For the DAB and trypan blue staining, the tobacco (*N. benthamiana*) leaf was infiltrated of *AhRRS5* in a small syringe with 1.0 cm diameter, the volume was about 100 μL. For the electrolyte leakage analysis, the second leaf was infiltrated with about 1 mL agrobacterium until spread to the whole leaf. The infiltrated leaves were harvested at the indicated time points for future analysis. Three biological replicates were set for the experiment.

### Tobacco transformation

*N. tabacum* cv. CB‐1, cv. Honghuadajinyuan were used as the host, and p35S::AhRRS5 fusion gene was transformed by the leaf‐disc method mediated by EHA105 to generate transgenic plants (Rizhsky *et al*., 2002). The initial transgenic T_0_ and T_1_ offspring were selected by kanamycin and confirmed by RT‐PCR to verify transgene integration. The T_2_ transgenic homozygous lines were obtained and used in this study.

### Quantitative real‐time RT‐PCR

Total RNA was extracted from peanut, transgenic tobacco and wild‐type seedlings through CTAB extraction (Chen *et al*., 2015). Reverse transcription was performed with PrimeScript™ RTase (TaKaRa, Dalian, China) in accordance with the manufacturer's instructions. Real‐time PCR for the relative expression level of target genes was performed with specific primers (see Table S1 for gene‐specific primers) essentially provided for the Master cyclereprealplex (Eppendorf, Hamburg, Germany) and SYBR Premix Ex Taq II (Perfect Real Time; TaKaRa, Dalian China). Each reaction mix (20 μL) contained 10 μL of SYBR Premix ExTaq (2×), 0.2 μL of PCR forward/reverse gene‐specific primers (10 μm) and diluted cDNA (2 μL). Three experimental replicates were performed for each gene using different cDNAs synthesized from three biological replicates. The PCR programme was as follows: 95 °C for 5 min; 40 cycles of 5 s at 95 °C, 30 s at 60 °C and 30 s at 72 °C; and 95 °C for 15 s, 60 °C for 1 min, 95 °C for 15 s and 60 °C for 15 s. The specificity of amplification was confirmed by melting curve analysis after 40 cycles. The relative expression level of the target gene was calculated using the comparative CT method (2^−ΔΔCT^ method) (Schmittgen and Livak, 2008) by normalizing the PCR threshold cycle number (Ct value) of the target gene with that of the reference gene. The Ct value was calculated as follows: ΔΔCt = (CT_gene_−CT_actin_) _treat_−(CT_gene_−CT_actin_)_control_. *Ahactin* was used as an internal reference to detect the relative transcript level of *AhRRS5* under different treatments in peanut. Tobacco *NtEF1*α was used as an internal reference to detect the relative transcript levels of related defence genes after treatment with *R. solanacearum* between the wild‐type and transgenic tobacco plants.

### Histochemical analysis and ion conductivity determination

Transient expression development was assessed 48 h after the transient overexpression of *AhRRS5* in tobacco leaves by staining the infected plants with 3, 3′‐diaminobenzidine (DAB; Sigma, St. Louis, MO) and lactophenol–ethanol–trypan blue. The infected tobacco leaves were incubated in 1 mg/mL DAB solution overnight at room temperature, boiled for 5 min in a solution of 3:1:1 ethanol/lactic acid/glycerol and then placed in absolute ethanol before observation to measure H_2_O_2_ level. Cell death was detected by boiling the inoculated leaves in trypan blue staining solution (10 mL of lactic acid, 10 mL of glycerol, 10 g of phenol, 30 mL of absolute ethanol and 10 mg of trypan blue, dissolved in 10 mL of ddH_2_O) for 2 min. The leaves were left at room temperature overnight, transferred into chloral hydrate solution (2.5 g of chloral hydrate dissolved in 1 mL of distilled water) and then boiled for 20 min to destain. The leaves were observed under a light microscope.

Ion conductivity was measured as previously described with minor modifications (Hwang and Hwang, 2011). Six round leaf discs (11 mm in diameter) per agroinfiltrated leave were cut, washed in ddH_2_O and then incubated in 20 mL of ddH_2_O with evacuation for 10 min at room temperature. Electrolyte leakage was measured using MettlerToledo 326.

### Microarray analysis

*In silico* analysis of *AhRRS5* gene expression pattern in peanut, microarray designing, hybridization, washing, and scanning and data analysis were performed as described by Chen *et al*. (2015). The gene expression intensity of all hybridizations was analysed, and expression levels were estimated among different tissues and under diverse stress conditions. The expression data of genes were normalized using quantile normalization (Bolstad *et al*., 2003) and generated using the Robust Multichip Average algorithm (Irizarry *et al*., 2003a,b). Three replicates were performed for all experiments.

Tobacco microarray analysis was performed using the leaves of the hyper‐resistant tobacco variety Yanyan 97, hypersusceptible tobacco variety Honghuadajinyuan, T_2_ generation transgenic tobacco of AhRRS5‐OE‐3, and wild‐type tobacco after *R. solanacearum* inoculation. Microarray designing, hybridization, washing, and scanning and data analysis were conducted as previously described (Zhang *et al*., 2016). Gene expression data were analysed as follows.

## Supporting information


**Table S1** Main primers for PCR used in this study.

**Table S2** Detailed data of disease indexes and death ratios of different OE lines and the wild type after inoculation with *Ralstonia solanacearum*.

**Data S1** Sequences of *AhRRS5* full‐length cDNA, genomic DNA and protein.

**Data S2** Amino acid sequences of four homologous R genes (list the function).

**Data S3** Twenty‐nine known functional R genes used for phylogenetic analysis.

**Data S4** *In silico* study of expression characteristics of four NBS‐LRR gene members in the *AhRRS5* family in peanut.

**Data S5** qPCR analysis of relative transcript levels of defence marker genes in leaves of T2 *AhRRS5‐OE‐3* lines compared to that in leaves of wild‐type tobacco plants.
