# Supplementary material for: Overexpression of a novel peanut NBS‐LRR gene AhRRS5 enhances disease resistance to Ralstonia solanacearum in tobacco
Source: Plant Biotechnol J. 2016 Jul 26;15(1):39–55. doi: 10.1111/pbi.12589 (PMC5253469; doi:10.1111/pbi.12589)
Supplement: Supplementary file 1 — Table S1 Main primers for PCR used in this study. [file PBI-15-39-s007.docx]

**Supplemental table 1 Main primers for PCR used in this study**

| **Primer name** | **Sequence（5’-3’）** |
| --- | --- |
| RACE-F | AAGCAGTGGTATCAACGCAGAGTGGCCAT |
| 3’ primer | ATTCTAGAGGCCGAGGCGGCCGACATGd(T)30N-1N-3'(N=A, G, C, or T; N-1=A, G, or C） |
| PRRS _1EW9_F | GCTTTGTAGAGGCAAATCAAGGCTG |
| PRRS _1EW9_R | TGAAGAGAAGGCATCCAATCAGGTAAG |
| AhRRS5- FL-F | CATATAACTAAGTGTGGGCCATTTCGAGAGG |
| AhRRS5- FL-R | TGGGTTTATTGAGCAATATTTACACTATTTAC |
| AhRRS5-BamH1-F | ATTAGGATCCACCATGGCTGAGAGTGCCATAGCCT |
| AhRRS5-Asc1-R | ATTAGGCGCGCCACACCTTTGAGAGAGTGCTGCGT |
| AhRRS5-OE-F | ATTAGGATCCACCATGGCTGAGAGTGCCATAGCCT |
| AhRRS5-OE-R | ATTTAGGCGCGCCTACACCTTTGAGAGAGTGCTGCGT |
| Ahactin-F | GAGGAGAAGCAGAAGCAAGTTG |
| Ahactin-R | AGACAGCATATCGGCACTCATC |
| AhRRS5-qRT-F | GCAGTGATGAACGCAGCACTCTCTC |
| AhRRS5-qRT-R | GTGCTGACATCTGTACAGGAAGTGAG |
| NtEF1ɑ-F | TGCTGCTGTAACAAGATGGATGC |
| NtEF1ɑ-R | GAGATGGGGA CAAAGGGGAT T |
| Ntactin-F | ATGAAGAACGTAGCGAAATG |
| Ntactin-R | GGGAATCCTCGTAAGTTTCT |
| NtHIN1-F | CGACCTAACAAAGTCAAGTTCTACG |
| NtHIN1-R | CTCTATCTCCCAATAAAACC AAGC |
| NtHSR201-F | CAGCAGTCCTTTGGCGTTGTC |
| NtHSR201-R | GCTCAGTTTAGCCGCAGTTGTG |
| NtHSR203-F | TGGCTCAACGATTACGCA |
| NtHSR203-R | GCACGAAACCTGGATGG |
| NtHSR515-F | TTGGGCAGAATAGATGGGTA |
| NtHSR515-R | TTTGGTGAAAGTCTTGGCTC |
| NtPR1a/c-F | AACCTTTGACCTGGGACGAC |
| NtPR1a/c-R | GCACATCCAACACGAACCGA |
| NtPR2-F | TGATGCCCTTTTGGATTCTATG |
| NtPR2-R | AGTTCCTGCCCCGCTTTCTATG |
| NtPR3-F | CAGGAGGGTATTGCTTTGTTAGG |
| NtPR3-R | CGTGGGAAGATGGCTTGTTGTC |
| NtPR4-F | GGAAAACGGAAAGGTAAGAAGAGG |
| NtPR4-R | GGACACGAGGTAGGTATCACAACAA |
| NtCHN50-F | ATGCCAAGGAAAGGGATTCTACA |
| NtCHN50-R | TGGGAGGTTTGGGCGAAGA |
| NtNPR1-F | GGCGAGGAGTCCGTTCTTTAAc |
| NtNPR1-R | TCAACCAGGAATGCCACAGC |
| NtPR1b-F | AACCCATCCATACTATTCCTTG |
| NtPR1b-R | GCCGCTAACCTATTGTCCC |
| NtACS6-F | GCATTGTTATGAGTGGAGGGG |
| NtACS6-R | CAGATTCTAAGGCTTCTTTTGTGAC |
| NtEFE26-F | CGGACGCTGGTGGCATAATC |
| NtEFE26-R | CAACAAGAGCTGGTGCTGGATA |
