# Supplementary material for: Overexpression of a novel peanut NBS‐LRR gene AhRRS5 enhances disease resistance to Ralstonia solanacearum in tobacco
Source: Plant Biotechnol J. 2016 Jul 26;15(1):39–55. doi: 10.1111/pbi.12589 (PMC5253469; doi:10.1111/pbi.12589)
Supplement: Supplementary file 3 — Data S1 Sequences of AhRRS5 full‐length cDNA, genomic DNA and protein. [file PBI-15-39-s005.docx]

**Data S1: Sequences of AhRRS5 full-length cDNA, genomic DNA and protein**

***AhRRS5* Full-length cDNA sequence：**

GAAAATAAAGAAAT**G**AAATTCATATAACTAAGTGTGGGCCATTTCGAGAGGAAAATCAGGACTAACAGAGAATATGTGACTAACCTGGAAAAGGAAACAATC**ATG**GCTGAGAGTGCCATAGCCTTTCTTCTCCAAAGATTAGTATCTGTGTTTGAGAATGAGGTGACATGGTTCCCAGGCATCCAAGAAGAAGTGGTTCACCTTAAAGGACATTTGGGGGTCATAAGAGCCTTCTTAAGAGTTGCAGATGCAAAACAAGAGAGTGACGAGGAACTCAAAGTTTGCATTAAGCAACTCAGAGACATTGCTCATGATGCTGAAGATCTTCTCGATGAACTAGAACTTGTCCAGGCATATGATCACACAAATGGATTCTCTGTTATTCTCAGTCGATTTTCTGGCCAAATCAGGCATATGAAAGCTCGCTATCGGATTGCTTCTGATTTAAAAGGCATCAACTCCCGCATGAGAACTATTTTGGGAGTCCTAGCTAAATTTGACACTGCTTCACAGGCTTCAAATTATACAGGTAAGGCATGGCATGATCAACGAGGGGATGCCCTTCTCCTGGAGAACACTGACCTAGTGGGTATAGAGGAGCCAAAAAAGCAGTTGATCAGTTGGTTGATCAAAGGATGCCCAGGGCGTAAAGTAATTTCTGTTACTGGTATGGGAGGGATGGGAAAAACCACTGTGGTGAAGAAAGTGTATGATGATCCAGAAGTAATAAAACACTTCAAAGCCTGTGTTTGGGTTACTGTTTCCCAGTCTTTTAAAACTGAGGAGCTTCTCAGAGACTTGGTCCAGAAAATCTTTTCTGAAATAAGAAGACCGGTTCCAGATGGCCTGGAAAGCATGAGGAGCGATAAGCTGAAGTTGATTATCAAGGACATGTTGCAAAGGAGGAGGTACCTGGTGGTATTTGATGATGTCTGGCATATGCATGAATGGGAAGCTGTCAAATATGCATTGCCTGACAATAACTGTGGCAGCAGGGTCATGATCACCACACGGAAATCTGATTTAGCCTCTGCCTGCAGCATACAATCCAAGGGTAAGGTGTATAACTTGCAACCCTTGAAAGAAGATGAAGTCTGGGATCTATTTACTAGAAAGACCTTTCAGGGAAAATCATGCCCCTCCTACTTGACCAGCATTTGTAAATGTATCTTAAGAAAGTGTGAAGGCTTACCCCTGGCAATTGTAGCAATCAGTAGTGTCCTGGCAATGAAGGACAAGTGCAGGATAGAAGAGTGGGATATGATTTGTCATAGTCTTGGTGCTGAAATTCAAGACAATGACAAACTTGGTAATTTGAAAACAGTACTTGGCCTCAGTATTAATGACTTGCCTTACTACTTAAAATACTGCTTCTTGTACTTGAGCATCTTTCCTGAGGACCATCTGATAGAGCGCATGAGATTGATTCGCTTATGGATAGCAGAAGGATTTATTGAAGCCAAAGAAGGCAAAACACTGGAAGATGTTGCAGAAGATTACCTCAAGGAGCTCCTGAACAGAAACTTAATACAAGTAGCAGGGACAACGACAGATGGAAGGGTCAAAACTTTGCGCATCCATGATCTCATACGGGAAATCATCATTTTGAAATCTAAGGATGAAAACTTTGCAACCATTGTCAAAGAACAAAGTGTGCCATGGCCCGAAAGGCTTCGACGCCTTTCAGTGCATAACACGATGCCCAATGGACAGCAACAGAGGTCTGTTTCTCAACTCCGTTCTCTTCTAATGTTTGGGGTTGCTGAACAGTTATCCTTATGCAAACTGTTTCCGGGAGGTTTTAGACTGCTTGCTGTTTTGGATTTTCAAGATGCACCTTTGCAGAAGTTTCCAGTAGCTATCGGTGGCCTATATTGTTTAAGGTATCTAAGCTTAAGGAATACAAAGGTGAATATGGTTCCTGGAAAAATATTAGGGAAGCTGAAGAACCTAGAAACACTGGATCTTAAGAAGACCTCCATCACAGAATTGCCTGCAGACATACTAAATCTTAAGAAACTTCGCCATCTCCTTGTGTATCAGGTTAAGGTCAAAGGTTATGGAGAGTTCCATTCTAAACTGGGTTTTAAAGCCCCCTCTGAAATAGGATACCTACAGTCATTACAAAAGCTTTGCTTTGTAGAGGCAAATCAAGGCTGTGGTAAGATCATTAGGCAGTTAGCGGAGCTATGTCAGTTAAGAAGGTTAGGCATCAGGAATCTGAGAGAGGAAGATGGCAAGGCTTTCTGTTTGTCCATTGAGAGGTTGGTCAATCTCTGTGCCCTCTCTGTTACCTCTGAGGGTGAGAATAAAGTCATTGCTCTAGAATTTCTTTCTTCACCCCCTCCATATCTGCAGCGCTTGTATTTGTCAGGACGCCTTCTAGACTTACCTGATTGGATGCCTTCTCTTCATAACCTGGCCAAGTTGTTTCTGAAATGGAGCTGTTTAGAACAAGATCCACTAGAATATCTGCAGGATTTGCCAAACCTTTCACATCTCGAATTACTTCAAGCATACACCGGCGACACATTGCATTTTCAATGTGGAAAGTTCAAGAAGCTCAAGATTCTAGGCCTTGACAGATTTGTTGAGCTAAAACAGGTGATTCTGGGGAAGGATGCAATGCCATGCCTAGAAAAGCTTATCATCCAGCGTTGCCAACTGTTGAAAAATGTGCCATCAGGCGTTGAACTCTTGACTAAGCTGAAAGTCCTGGAGCTTTTTGACATGCCTGATGAACTAATGAAGACAATATGTCCACAGGGTCCGGGAAAAGATTACTGGAAAGTTGCACATATACCAGAAGTCTTTTCTACCTACTGGAGAGACGGGGCTTGGGATGTCTACCCACTGGAAAGTTTCAAAGACTGTTCTCCACGGTCTGGCACTGTCATGCGCAGTGATGAACGCAGCACTCTCTCAAAGGTG**TAG**CTTTATATTAAACACCAGCAGCATGTAAATTCATGTAAATAACCTAAACTTTGTACATAGAAATAATTGTACAAAGTAAACCTCACTTCCTGTACAGATGTCAGCACTTTGAATAAAGGTGTAGGTTTTAATTCACCTTTGTATAATCTATGATTAAT**GTAAATAGTGTAAATATTGCTCAATAAACCCAC**TGTATATAATTCGACCAAAAAAAAAAAAAAAAAAAAAAAAAAAAAAA

**AhRRS5 protein sequence：**943aa

MAESAIAFLLQRLVSVFENEVTWFPGIQEEVVHLKGHLGVIRAFLRVADAKQESDEELKVCIKQLRDIAHDAEDLLDELELVQAYDHTNGFSVILSRFSGQIRHMKARYRIASDLKGINSRMRTILGVLAKFDTASQASNYTGKAWHDQRGDALLLENTDLVGIEEPKKQLISWLIKGCPGRKVISVTGMGGMGKTTVVKKVYDDPEVIKHFKACVWVTVSQSFKTEELLRDLVQKIFSEIRRPVPDGLESMRSDKLKLIIKDMLQRRRYLVVFDDVWHMHEWEAVKYALPDNNCGSRVMITTRKSDLASACSIQSKGKVYNLQPLKEDEVWDLFTRKTFQGKSCPSYLTSICKCILRKCEGLPLAIVAISSVLAMKDKCRIEEWDMICHSLGAEIQDNDKLGNLKTVLGLSINDLPYYLKYCFLYLSIFPEDHLIERMRLIRLWIAEGFIEAKEGKTLEDVAEDYLKELLNRNLIQVAGTTTDGRVKTLRIHDLIREIIILKSKDENFATIVKEQSVPWPERLRRLSVHNTMPNGQQQRSVSQLRSLLMFGVAEQLSLCKLFPGGFRLLAVLDFQDAPLQKFPVAIGGLYCLRYLSLRNTKVNMVPGKILGKLKNLETLDLKKTSITELPADILNLKKLRHLLVYQVKVKGYGEFHSKLGFKAPSEIGYLQSLQKLCFVEANQGCGKIIRQLAELCQLRRLGIRNLREEDGKAFCLSIERLVNLCALSVTSEGENKVIALEFLSSPPPYLQRLYLSGRLLDLPDWMPSLHNLAKLFLKWSCLEQDPLEYLQDLPNLSHLELLQAYTGDTLHFQCGKFKKLKILGLDRFVELKQVILGKDAMPCLEKLIIQRCQLLKNVPSGVELLTKLKVLELFDMPDELMKTICPQGPGKDYWKVAHIPEVFSTYWRDGAWDVYPLESFKDCSPRSGTVMRSDERSTLSKV

**P-LOOP: GMGGMGKTTV**

**RNBS-A-nonTIR: FKACVWVTVSQS**

**Kinase-2a: LQRRRYLVVFDDVW**

**Kinase-3a: GSRVMITTR**

**RNBS-C: LQPLKEDEVWDLFTRKTF**

**GLPL: GLPL**

**LRR: CLRYLSLRNTKVNMVPGKILGKLKNL**

**LRR: QLAELCQLRRLGIRNLREEDGKAFCLSI**

**LRR: NLAKLFLKWSCLEQDPLEYLQDLPNLSHL**

**NSL：GKFKKLKILGLDRF**

***AhRRS5* gDNA sequence：there is an antron within the genome sequence of *AhRRS5 (blue letters)***

**GAAATTCATATAACTAAGTGTGGGCCAT**TTCGAGAGGAAAATCAGGACTAACAGAGAATATGTGACTAACCTGGAAAAGGAAACAATC**ATG**GCTGAGAGTGCCATAGCCTTTCTTCTCCAAAGATTAGTATCTGTGTTTGAGAATGAGGTGACATGGTTCCCAGGCATCCAAGAAGAAGTGGTTCACCTTAAAGGACATTTGGAGGTCATAAGAGCCTTCTTAAGAGTTGCAGATGCAAAACAAGAGAGTGACGAGGAACTCAAAGTTTGCATTAAGCAACTCAGAGACATTGCTCATGATGCTGAAGATCTTCTCGATGAACTAGAACTTGTCCAGGCATATGATCACACAAATGGATTCTCTGTTATTCTCAGTCGATTTTCTGGCCAAATCAGGCATATGAAAGCCCGCTATCGGATTGCTTCTGATTTAAAAGGCATCAACTCCCGCATGAGAACTATTTTGGGAGTCCTAGCTAAATTTGACACTGCTTCACAGGCTTCAAATTATACAG***GT****AGAACTAATTCTGCCACTCTTTCAAGCTAAAGTATTGACTGTTGAGTGTCCTAAAGCTAAGGCTCAGGACTTCCAAATATGCTTTTTCCCTTTTTTTGATAAAATCAGTTATGCTATTTTTAATTCTACATATCTATTTTCCTTGGGTTCTAGCTGACTATTAGCTAGATTTATAGTCAGTGAGCTTCTGCTACTTTCTAGTTGAAGCTTTTCACTATACTTATTTGTAACACATGCTAAACTGTTCTCTTTAGTCTTTCAGGGGTTGTTTGTGTGTTGAGGATTCAGAGTGGGAGGGAAGGGAAAGATTTGCAGAGAAATTGACTAATAATTACTCTGCAGACCTTCCCTTTTCATTTTAAAACCTCGTGTCATAAACATGCCTTATATTACTACTACTACTACAGAATAGATCGATCTATAAATGCTTCCCCTAGACCAAAAGCTTCATCTGATATATTATAGATACCATCAATTGGCCATGATATACTTTTCCTTTGTGAGAATGGTATAATAAATATATTCTTAAAC****AG***GTAAGGCATGGCATGATCAACGAGGGGATGCCCTTCTCCTGGAGAACACTGACCTAGTGGGTATAGAGGAGCCAAAAAAGCAGTTGATCAGTTGGTTGATCAAAGGATGCCCAGGGCGTAAAGTAATTTCTGTTACTGGTATGGGAGGGATGGGAAAAACCACTGTGGTGAAGAAAGTGTATGATGATCCAGAAGTAATAAAACACTTCAAAGCCTGTGTTTGGGTTACTGTTTCCCAGTCTTTTAAAACTGAGGAGCTTCTCAGAGACTTGGTCCAGAAAATCTTTTCTGAAATAAGAAGACCGGTTCCAGATGGCCTGGAAAGCATGAGGAGCGATAAGCTGAAGTTGATTATCAAGGACATGTTGCAAAGGAGGAGGTACCTGGTGGTATTTGATGATGTCTGGCATATGCATGAATGGGAAGCTGTCAAATATGCATTGCCTGACAATAACTGTGGCAGCAGGGTCATGATCACCACACGGAAATCTGATTTAGCCTCTGCCTGCAGCATACAATCCAAGGGTAAGGTGTATAACTTGCAACCCTTGAAAGAAGATGAAGTCTGGGATCTATTTACTAGAAAGACCTTTCAGGGAAAATCATGCCCCTCCTACTTGACCAGCATTTGTAAATGTATCTTAAGAAAGTGTGAAGGCTTACCCCTGGCAATTGTAGCAATCAGTGGTGTCCTGGCAATGAAGGACAAGTGCAGGATAGAAGAGTGGGATATGATTTGTCGTAGTCTTGGTGCTGAAATTCAAGACAATGACAAACTTGGTAATTTGAAAACAGTACTTGGCCTCAGTATTAATGATTTGCCTTACTACTTAAAATACTGCTTCTTGTACTTGAGCATCTTTCCTGAGGACCATCTGATAGAGCGCATGAGATTGATTCGCTTATGGATAGCAGAAGGATTTATTGAAGCCAAAGAAGGCAAAACACTGGAAGATGTTGCAGAAGATTACCTCAAGGAGCTCCTGAACAGAAACTTAATACAAGTAGCAGGGACAACGACAGATGGAAGGGTCAAAACTTTGCGCATCCATGATCTCATACGGGAAATCATCATTTTGAAATCTAAGGATGAAAACTTTGCAACCATTGTCAAAGAACAAAGTGTGCCATGGCCCGAAAGGCTTCGACGCCTTTCAGTGCATAACACGATGCCCAATGGACAGCAACAGAGGTCTGTTTCTCAACTCCGTTCTCTTCTAATGTTTGGGGTTGCTGAACAGTTATCCTTATGCAAACTGTTTCCGGGAGGTTTTAGACTGCTTGCTGTTTTGGATTTTCAAGATGCACCTTTGCAGAAGTTTCCAGTAGCTATCGGTGGCCTATATTGTTTAAGGTATCTAAGCTTAAGGAATACAAAGGTGAATATGGTTCCTGGAAAAATATTAGGGAAGCTGAAGAACCTAGAAACACTGGATCTTAAGAAGACTTCCATCACAGAATTGCCTGCAGACATACTAAATCTTAAGAAACTTCGCCATCTCCTTGTGTATCAGGTTAAGGTCAAAGGTTATGGAGAGTTCCATTCTAAACTGGGTTTTAAAGCCCCCTCTGAAATAGGATACCTACAGTCATTACAAAAGCTTTGCTTTGTAGAGGCAAATCAAGGCTGTGGTAAGATCATTAGGCAGTTAGCGGAGCTATGTCAGTTAAGAAGGTTAGGCATCAGGAATCTGAGAGAGGAAGATGGCAAGGCTTTCTGTTTGTCCATTGAGAGGTTGGTCAATCTCTGTGCCCTCTCTGTTACCTCTGAGGGTGAGAATAAAGTCATTGCTCTAGAATTTCTTTCTTCACCCCCTCCATATCTGCAGCGCTTGTATTTGTCAGGACGCCTTCTAGACTTACCTGATTGGATGCCTTCTCTTCATAACCTGGCCAAGTTGTTTCTGAAATGGAGCTGTTTAGAACAAGATCCACTAGAATATCTGCAGGATTTGCCAAACCTTTCACATCTCGAATTACTTCAAGCATACACCGGCGACACATTGCATTTTCAATGTGGAAAGTTCAAGAAGCTCAAGATTCTAGGCCTTGACAGATTTGTTGAGCTAAAACAGGTGATTCTGGGGAAGGATGCAATGCCATGCCTAGAAAAGCTTATCATCCAGCGTTGCCAACTGTTGAAAAATGTGCCATCAGGCGTTGAACTCTTGACTAAGCTGAAAGTCCTGGAGCTTTTTGACATGCCTGATGAACTAACGAAGACAATATGTCCACAGGGTCCGGGAAAAGATTACTGGAAAGTTGCACATATACCAGAAGTCTTTTCTACCTACTGGAGAGACGGGGCTTGGGATGTCTACCCACTGGAAAGTTTCAAAGACTGTTCTCCACGGTCTGGCACTGTCATGCGCAGTGATGAACGCAGCACTCTCTCAAAGGTG**TAG**CTTTATATTAAACACCAGCAGCATGTAAATTCATGTAAATAACCTAAACTTTGTACATAGAAATAATTGTACAAAGTAAACCTCACTTCCTGTACAGATATCAGCACTCTGAATAAAGGTGTAGGTTTTAATTCACCTTTGTATAATCTATGATTAATGTAAATAGTGTAAATATTGCT**CAATAAACCCACTGTATATAATTCGACC**
