# Supplementary material for: Overexpression of a novel peanut NBS‐LRR gene AhRRS5 enhances disease resistance to Ralstonia solanacearum in tobacco
Source: Plant Biotechnol J. 2016 Jul 26;15(1):39–55. doi: 10.1111/pbi.12589 (PMC5253469; doi:10.1111/pbi.12589)
Supplement: Supplementary file 4 — Data S2 Amino acid sequences of four homologous R genes (list the function). [file PBI-15-39-s001.docx]

Data S2: Amino acide sequences of four homolog R-genes (list the function)

>OsPid3

MAEGVVGSLIVKLGDALASEAVEVAKSLLGLEGSALKRLFSEIGEVKGELESIHAFLQAAERFKDADETTSAFVKQVRSLALSIEDVVDEFTYELGEGDGRMGMAVALKRMCKMGTWSRLAGNLQDIKVNLKNAAERRIRYDLKGVERGAKSTAGRRSSNWRSDSVLFKREDELVGIEKKRDLLMKWVKDEEQRRMVVSVWGMGGIGKTALVANVYNAIKADFDTCAWITVSQSYEADDLLRRTAQEFRKNDRKKDFPVDVDITNYRGLVETTRSYLENKRYVLVLDDVWNANVWFDSKDAFEDGNIGRIILTSRNYDVALLAHETHIINLQPLEKHHAWDLFCKEAFWKNEIRNCPPELQPWANNFVDKCNGLPIAIVCIGRLLSFQGSTYSDWEKVYKNLEMQLTNNSIMDMMNIILKISLEDLPHNIKNCFLYCSMFPENYVMKRKSLVRLWVAEGFIEETEHRTLEEVAEHYLTELVNRCLLLLVKRNEAGHVHEVQMHDILRVLALSKAREQNFCIVVNHSRSTHLIGEARRLSIQRGDFAQLADHAPHLRSLLLFQSSPNVSSLHSLPKSVKLLSVLDLTDSSVDRLPKEVFGLFNLRFLGLRRTKISKLPSSIGRLKNLLVLDAWKCKIVKLPLAITKLQKLTHLIVTSKAVVVSKQFVPSVGVPAPLRICSMTTLQTLLLMEASSQMVHHLGSLVELRTFRISKVRSCHCEQLFMAITNMIHLTRLGIQADSSQEVLHLESLKPPPLLQKLFLQGTLSHESLPHFVSVSNLNNLTFLRLAGSRIDENAFLNLEGLQQLVKLQLYDAFDGMNIYFHENSFPKLRILKIWGAPHLNEIKMTKGAVASLTHLKFLLCPNLKQLPCGIEHVRTLEELTLDHTAEELVDRVRRKKERMICDVQRVYVGFIRNGVLAAERIQ

>AtRPM1

MASATVDFGIGRILSVLENETLLLSGVHGEIDKMKKELLIMKSFLEDTHKHGGNGSTTTTTQLFQTFVANTRDLAYQIEDILDEFGYHIHGYRSCAKIWRAFHFPRYMWARHSIAQKLGMVNVMIQSISDSMKRYYHSENYQAALLPPGDAKWVNNISESSLFFSENSLVGIDAPKGKLIGRLLSPEPQRIVVAVVGMGGSGKTTLSANIFKSQSVRRHFESYAWVTISKSYVIEDVFRTMIKEFYKEADTQIPAELYSLGYRELVEKLVEYLQSKRYIVVLDDVWTTGLWREISIALPDGIYGSRVMMTTRDMNVASFPYGIGSTKHEIELLKEDEAWVLFSNKAFPASLEQCRTQNLEPIARKLVERCQGLPLAIASLGSMMSTKKFESEWKKVYSTLNWELNNNHELKIVRSIMFLSFNDLPYPLKRCFLYCSLFPVNYRMKRKRLIRMWMAQRFVEPIRGVKAEEVADSYLNELVYRNMLQVILWNPFGRPKAFKMHDVIWEIALSVSKLERFCDVYNDDSDGDDAAETMENYSSRHLCIQKEMTPDSIRATNLHSLLVCSSAKHKMELLPSLNLLRALDLEDSSISKLPDCLVTMFNLKYLNLSKTQVKELPKNFHKLVNLETLNTKHSKIEELPLGMWKLKKLRYLITFRRNDGHDSNWNYVLGTRVVPKIWQLKDLQVMDCFNAEDELIKNLGCMTQLTRISLVMVRREHGRDLCDSLNKIKRIRFLSLTSIDEEEPLEIDDLIATASIEKLFLAGKLERVPSWFNTLQNLTYLGLRGSQLQENAILSIQTLPRLVWLSFYNAYMGPRLRFAQGFQNLKILEIVQMKHLTEVVIEDGAMFELQKLYVRACRGLEYVPRGIENLINLQELHLIHVSNQLVERIRGEGSVDRSRVKHIPAIKHYFRTDNGSFYVSLSS

>ZmRXO1

MAEIAVLLVLKKIAIALAGETLSFAKPLLAKKSESVAALPDDMKLISNELELIRAFLKEIGRKGWKSEVIETWIGQVRRLAYDMEDTVDHFIYVVGTHDQMGSCWDYMKKIAKKPRRLVSLDEIASEIKKIKQELKQLSESRDRWTKPLDGGSGIPAGSYETEKEMYLPGHDYTISDEELAGIDENKQTLISSLKFEDPSLRIIAVWGMGGVGKSTLVNNVYKNEGSNFDCRAWVSISQSYRLEDIWKKMLTDLIGKDKIEFDLGTMDSAELREQLTKTLDKRQYLIILDDVWMANVFFKIKEVLVDNGLGSRVIITTRIEEVASLAKGSCKIKVEPLGVDDSWHVFCRKAFLKDENHICPPELRQCGINIVEKCDGLPLALVAIGSILSLRPKNVDEWKLFYDQLIWELHNNENLNRVEKIMNLSYKYLPDYLKNCFLYCAMFPEDYLIHRKRLIRLWIAEGFIEQKGACSLEDTAESYLKELIRRSMLHVAERNCFGRIKCIRMHDLVRELAIFQSKREGFSTTYGGNNEAVLVGSYSRRVAVLQCSKGIPSTIDPSRLRTLITFDTSRALSVWYSSISSKPKYLAVLDLSSLPIETIPNSIGELFNLRLLCLNKTKVKELPKSITKLQNLQTMSLENGELVKFPQGFSKLKKLRHLMVSRLQDVTFSGFKSWEAVEPFKGLWTLIELQTLYAITASEVLVAKLGNLSQLRRLIICDVRSNLCAQLCGSLSKLCQLSRLTIRACNEDEVLQLDHLTFPNPLQTLSLDGRLSEGTFKSPFFLNHGNGLLRLMLFYSQLSENPVPHLSELSNLTRLSLIKAYTGQELYFQAGWFLNLKELYLKNLSRLNQIDIQEGALASLERITMKHLPELREVPVGFRFLKSLKTIFFSDMHPEFESSFQKEM

>AhRRS5

MAESAIAFLLQRLVSVFENEVTWFPGIQEEVVHLKGHLGVIRAFLRVADAKQESDEELKVCIKQLRDIAHDAEDLLDELELVQAYDHTNGFSVILSRFSGQIRHMKARYRIASDLKGINSRMRTILGVLAKFDTASQASNYTGKAWHDQRGDALLLENTDLVGIEEPKKQLISWLIKGCPGRKVISVTGMGGMGKTTVVKKVYDDPEVIKHFKACVWVTVSQSFKTEELLRDLVQKIFSEIRRPVPDGLESMRSDKLKLIIKDMLQRRRYLVVFDDVWHMHEWEAVKYALPDNNCGSRVMITTRKSDLASACSIQSKGKVYNLQPLKEDEVWDLFTRKTFQGKSCPSYLTSICKCILRKCEGLPLAIVAISSVLAMKDKCRIEEWDMICHSLGAEIQDNDKLGNLKTVLGLSINDLPYYLKYCFLYLSIFPEDHLIERMRLIRLWIAEGFIEAKEGKTLEDVAEDYLKELLNRNLIQVAGTTTDGRVKTLRIHDLIREIIILKSKDENFATIVKEQSVPWPERLRRLSVHNTMPNGQQQRSVSQLRSLLMFGVAEQLSLCKLFPGGFRLLAVLDFQDAPLQKFPVAIGGLYCLRYLSLRNTKVNMVPGKILGKLKNLETLDLKKTSITELPADILNLKKLRHLLVYQVKVKGYGEFHSKLGFKAPSEIGYLQSLQKLCFVEANQGCGKIIRQLAELCQLRRLGIRNLREEDGKAFCLSIERLVNLCALSVTSEGENKVIALEFLSSPPPYLQRLYLSGRLLDLPDWMPSLHNLAKLFLKWSCLEQDPLEYLQDLPNLSHLELLQAYTGDTLHFQCGKFKKLKILGLDRFVELKQVILGKDAMPCLEKLIIQRCQLLKNVPSGVELLTKLKVLELFDMPDELMKTICPQGPGKDYWKVAHIPEVFSTYWRDGAWDVYPLESFKDCSPRSGTVMRSDERSTLSKV

>GmRPM1-like

MAESAVSFLLERLKPVFVNKLKLFTGVEAEVIYLKGQLELIRAFLRAADAFEESDEELKVWVRQVRDVVHEAEDLLDELELVQLHNHTNGLSNYLSIRNMKAHYRIAHELKAINSRMKTISLTRKRFLSKLDTASEASNSTYTVNAWHDQRGDALLLDNTDLVGIDRPKKQLIGWLINGCTGRKVISVTGMGGMGKTTLVKKVFDDPEVRKHFKACVWVTVSQSCKTEELLRDLARKLFSEIRRPIPEGLESMCSDKLKMIIKDLLQRKRYLVVFDDVWQMYEWEAVKYALPNNNCGSRIMITTRKSNLAFTSSIESNGKVYNLQPLKEDEAWDLFCRNTFQGHSCPSHLIDICKYILRKCGGLPLAIVAISGVLATKDKHRIDEWDMICRSLGAEIQGNGKLDNFKTVLNLSFNDLPYHLKYCFLYLSIFPEDYLIQRMRLIRLWIAEGFIKAKEGKTKEDVADDYLKELLNRNLIQVAEITSDGRVKTLRIHDLLREIIILKSKDQNFVSVVKEQSIAWPEKIRRLSVHGTLPCHRQQHIHRSGSQLRSLLMFGVGENLSLGKLFPGGCKLLGVLDYQDAPLNKFPVAVVDLYHLRYLSLRNTKVTMVPGYIIGKLHNLETLDLKKTSVRELPLDILKLQKLRHLLVYKFNVKGYAQFYSKHGFKAPTEIGNLKALQKLCFVEANQDCGMIIRQLGELSQLRRLGILKLREEDGKAFCLSIERLTNLHALSVASEGENKVIDLAFLCSPPPFLQRLYLSGRLQELPSWIQSLHSLARLFLKWSCLKHDPLVYLQDLPSLAHLELVQVYDGDTLHFVCGKFKKLKVLGLDKFDGLKQVTVGEDAMPCLERLSIGRCELLKKVPSGIEHLSKLKVLEFFDMPDELMKTICPHGPGKDYCKVSHIPNVYSTYWRDDGWDVYALDSFSRDCSPRSGTVMRSHEPRTLWKV
