# Supplementary material for: Overexpression of a novel peanut NBS‐LRR gene AhRRS5 enhances disease resistance to Ralstonia solanacearum in tobacco
Source: Plant Biotechnol J. 2016 Jul 26;15(1):39–55. doi: 10.1111/pbi.12589 (PMC5253469; doi:10.1111/pbi.12589)
Supplement: Supplementary file 5 — Data S3 Twenty‐nine known functional R genes used for phylogenetic analysis. [file PBI-15-39-s002.docx]

Data S3: Twenty-nine known functional R-genes used for phylogenesis analysis

>RPP1[Arabidopsis thaliana]

MSVMSLGCSKRKATNQDVDSESRKRRKICSTNDAENCRFIQDESSWKHPWSLCANRVISVAAVALTNFRFQQDNQESNSSSLSLPSPATSVSRNWKHDVFPSFHGADVRRTFLSHIMESFRRKGIDTFIDNNIERSKSIGPELKEAIKGSKIAIVLLSRKYASSSWCLDELAEIMKCRQMVGQIVMTIFYEVDPTDIKKQTGEFGKAFTKTCRGKPKEQVERWRKALEDVATIAGYHSHSWRNEADMIEKISTDVSNMLNSFTPSRDFDGLVGMRAHMDMLEQLLRLDLDEVRMIGIWGPPGIGKTTIARFLFNQVSDRFQLSAIMVNIKGCYPRPCFDEYSAQLQLQNQMLSQMINHKDIMISHLGVAQERLRDKKVFLVLDEVDQLGQLDALAKETRWFGPGSRIIITTEDLGVLKAHGINHVYKVEYPSNDEAFQIFCMNAFGQKQPHEGFDEIAWEVTCLAGELPLGLKVLGSALRGKSKREWERTLPRLKTSLDGKIGSIIQFSYDVLCDEDKYLFLYIACLFNGESTTKVKELLGKFLDVKQGLHLLAQKSLISFDGERIHMHTLLEQFGRETSRKQFVHHGFTKRQLLVGARGICEVLDDDTTDSRRFIGIHLELSNTEEELNISEKVLERVHDFHFVRIDASFQPERLQLALQDLIYHSPKIRSLNWYGYESLCLPSTFNPEFLVELDMRSSNLRKLWEGTKQLRNLKWMDLSYSSYLKELPNLSTATNLEELKLRNCSSLVELPSSIEKLTSLQILDLENCSSLEKLPAIENATKLRELKLQNCSSLIELPLSIGTATNLKQLNISGCSSLVKLPSSIGDITDLEVFDLSNCSSLVTLPSSIGNLQNLCKLIMRGCSKLEALPININLKSLDTLNLTDCSQLKSFPEISTHISELRLKGTAIKEVPLSIMSWSPLADFQISYFESLMEFPHAFDIITKLHLSKIQEVPPWVKRMSRLRDLSLNNCNNLVSLPQLSDSLDYIYADNCKSLERLDCCFNNPEIRLYFPKCFKLNQEARDLIMHTCIDAMFPGTQVPACFIHRATSGDSLKIKLKESPLPTTLRFKACIMLVKVNEELMSYDQTPMIVDIVIRDEHNDLKEKIYPSIYPSIYPLLTEHIYTFELDVEEVTSTELVFEFPQLNKRNWKIGECGILQRETRSLRRSSSPDLSPESSRVSSYDHCLRGD

>RPP5[Arabidopsis thaliana]

MAASSSSGRRRYDVFPSFSGVDVRKTFLSHLLKALDGKSINTFIDHGIERSRTIAPELISAIREARISIVIFSKNYASSTWCLNELVEIHKCFNDLGQMVIPVFYDVDPSEVRKQTGEFGKVFEKTCEVSKDKQPGDQKQRWVQALTDIANIAGEDLLNGPNEAHMVEKISNDVSNKLITRSKCFDDFVGIEAHIEAIKSVLCLESKEARMVGIWGQSGIGKSTIGRALFSQLSSQFHHRAFLTYKSTSGSDVSGMKLSWQKELLSEILGQKDIKIEHFGVVEQRLNHKKVLILLDDVDNLEFLKTLVGKAEWFGSGSRIIVITQDRQLLKAHEIDLVYEVKLPSQGLALKMISQYAFGKDSPPDDFKELAFEVAELVGSLPLGLSVLGSSLKGRDKDEWVKMMPRLRNDSDDKIEETLRVGYDRLNKKNRELFKCIACFFNGFKVSNVKELLEDDVGLTMLAEESLIRITPVGYIEMHNLLEKLGREIDRAKSKGNPGKRQFLTNFEDIREVLTEKTGTETLLGIRLPHPGYLTTRSFLIDEKSFKGMRNLQYLEIGYWSDGVLPQSLVYFPRKLKRLWWDNCPLKRLPSNFKAEYLVELRMVNSKLEKLWDGTQPLGSLKKMDLYNSYKLKEIPDLSLAINLEELNLEECESLETLPSSIQNAIKLRELNCWGGLLIDLKSLEGMCNLEYLSVPSWSSRECTQGIVYFPRKLKSVLWTNCPLKRLPSNFKAEYLVELIMEYSELEKLWDGTQSLGSLKEMNLRYSNNLKEIPDLSLAINLEELDLFGCVSLVTLPSSIQNATKLIYLDMSECENLESFPTVFNLKSLEYLDLTGCPNLRNFPAIKMGCAWTRLSRTRLFPEGRNEIVVEDCFWNKNLPAGLDYLDCLMRCMPCEFRSEQLTFLNVSGCKLEKLWEGIQSLGSLEEMDLSESENLKELPDLSKATNLKLLCLSGCKSLVTLPSTIGNLQNLRRLYMNRCTGLEVLPTDVNLSSLETLDLSGCSSLRTFPLISTNIVCLYLENTAIEEIPDLSKATKLESLILNNCKSLVTLPSTIGNLQNLRRLYMNRCTGLELLPTDVNLSSLETLDLSGCSSLRTFPLISTRIECLYLENTAIEEVPCCIEDFTRLTVLRMYCCQRLKNISPNIFRLTSLTLADFTDCRGVIKALSDATVVATMEDHVSCVPLSENIEYTCERFWDACSDYYSDDFEVNRNPIRLSTMTVNDVEFKFCCSITIKECGVRLLYVYQETEHNQQTTRSKKRMRMTSGTSEEDINLPYGQIVADTGLAALNTELSLGQGEASSSTSLEGEALCVDDYMINEEQDEQIPILYPVYDIDDDMWRSLFFGDTDDDMWRSLYSAE

>RPP4[Arabidopsis thaliana]

MASSSSSPSSRRYDVFPSFSGVDVRKTFLSHLIEALDRRSINTFMDHGIVRSCIIADALITAIREARISIVIFSENYASSTWCLNELVEIHKCYKKGEQMVIPVFYGVDPSHVRKQIGGFGDVFKKTCEDKPEDQKQRWVKALTDISNLAGEDLRNGPTEAFMVKKIANDVSNKLFPLPKGFGDFVGIEDHIKAIKSILCLESKEARIMVGIWGQSGIGKSTIGRALFSQLSSQFHHRAFITYKSTSGSDVSGMKLSWEKELLSEILGQKDIKIDHFGVVEQRLKHKKVLILLDDVDNLEFLKTLVGKAEWFGSGSRIIVITQDKQLLKAHEIDLVYEVELPSQGLALKMISQYAFGKDSPPDDFKELAFEVAELVGSLPLGLSVLGSSLKGRDKDEWVKMMPRLRNDSDDKIEETLRVGYDRLNKKNRELFKCIACFFNGFKVSNVKELLEDDVGLTMLADKSLIRITPDGDIEMHNLLEKLGREIDRAKSKGNPAKRQFLTNFEDIQEVVTEKTGTETVLGIRVPPTVLFSTRPLLVINEESFKGMRNLQYLEIGHWSEIDLPQGLVYLPLKLKLLKWNYCPLKSLPSTFKAEYLVNLIMKYSKLEKLWEGTLPLGSLKKMDLGCSNNLKEIPDLSLAINLEELNLSKCESLVTLPSSIQNAIKLRTLYCSGVLLIDLKSLEGMCNLEYLSVDWSSMEGTQGLIYLPRKLKRLWWDYCPVKRLPSNFKAEYLVELRMENSDLEKLWDGTQPLGSLKEMYLHGSKYLKEIPDLSLAINLERLYLFGCESLVTLPSSIQNATKLINLDMRDCKKLESFPTDLNLESLEYLNLTGCPNLRNFPAIKMGCSYFEILQDRNEIEVEDCFWNKNLPAGLDYLDCLMRCMPCEFRPEYLTFLDVSGCKHEKLWEGIQSLGSLKRMDLSESENLTEIPDLSKATNLKRLYLNGCKSLVTLPSTIGNLHRLVRLEMKECTGLELLPTDVNLSSLIILDLSGCSSLRTFPLISTRIECLYLENTAIEEVPCCIEDLTRLSVLLMYCCQRLKNISPNIFRLTSLMVADFTDCRGVIKALSDATVVATMEDHVSCVPLSENIEYTCERFWDELYERNSRSIFSYKDEDGDVYWVNWDLMMMLMLI

>RRS1-R[Arabidopsis thaliana] ADM88042.1

MTNCEKDEEFVCISCVEEVRYSFVSHLSEALRRKGINNVVVDVDIDDLLFKESQAKIEKAGVSVMVLPGNCDPSEVWLDKFAKVLECQRNNKDQAVVSVLYGDSLLRDQWLSELDFRGLSRIHQSRKECSDSILVEEIVRDVYETHFYVGRIGIYSKLLEIENMVNKQPIGIRCVGIWGMPGIGKTTLAKAVFDQMSSAFDASCFIEDYDKSIHEKGLYCLLEEQLLPGNDATIMKLSSLRDRLNSKRVLVVLDDVCNALVAESFLEGFDWLGPGSLIIITSRDKQVFRLCGINQIYEVQGLNEKEARQLFLLSASIMEDMGEQNLHELSVRVISYANGNPLAISVYGRELKGKKKLSEMETAFLKLKRRPPFKIVDAFKSSYDTLSDNEKNIFLDIACFFQGENVNYVIQLLEGCGFFPHVEIDVLVDKCLVTISENRVWLHKLTQDIGREIINGETVQIERRRRLWEPWSIKYLLEYNEHKANGEPKTTFKRAQGSEEIEGLFLDTSNLRFDLQPSAFKNMLNLRLLKIYCSNPEVHPVINFPTGSLHSLPNELRLLHWENYPLKSLPQNFDPRHLVEINMPYSQLQKLWGGTKNLEMLRTIRLCHSQHLVDIDDLLKAENLEVIDLQGCTRLQNFPAAGRLLRLRVVNLSGCIKIKSVLEIPPNIEKLHLQGTGILALPVSTVKPNHRELVNFLTEIPGLSEASKLERLTSLLESNSSCQDLGKLICLELKDCSCLQSLPNMANLDLNVLDLSGCSSLNSIQGFPRFLKQLYLGGTAIREVPQLPQSLEILNAHGSCLRSLPNMANLEFLKVLDLSGCSELETIQGFPRNLKELYFAGTTLREVPQLPLSLEVLNAHGSDSEKLPMHYKFNNFFDLSQQVVNDFFLKALTYVKHIPRGYTQELINKAPTFSFSAPSHTNQNATFDLQPGSSVMTRLNHSWRNTLVGFGMLVEVAFPEDYCDATDVGISCVCRWSNKEGRSCRIERNFHCWAPGKVVPKVRKDHTFVFSDVNMRPSTGEGNDPDIWAGLVVFEFFPINQQTKCLNDRFTVTRCGVRVINVATGNTSLENISLVLSLDPVEVSGYEVLRVSYDDLQEMDKVLFLYIASLFNDEDVDFVAPLIAGIDLDVSSGLKVLADVSLISVSSNGEIVMHSLQRQMGKEILHGQSMLLSDCESSMTENLSDVPKKEKKHRESKVKKVVSIPAIDEGDLWTWRKYGQKDILGSRFPRGYYRCAYKFTHGCKATKQVQRSETDSNMLAITYLSEHNHPRPTKRKALADSTRSTSSSICSAITTSASSRVFQNKDEPNQPHLPSSSTPPRNAAVLFKMTDMEEFQDNMEVDNDVVDTRTLALFPEFQHQPEEEDPWSTFFDDYNFYF

>RLM3[Arabidopsis thaliana] AEE83835.1

MLENIAKDVSNKLFPPSNNFSDFVGIEAHIEALISMLRFDSKKARMIGICGPSETGKTTIGRALYSRLKSDFHHRAFVAYKRKIRSDYDQKLYWEEQFLSEILCQKDIKIEECGAVEQRLKHTKVLIVLDDVDDIELLKTLVGRIRWFGSESKIVVITQKRELLKAHNIAHVYEVGFPSEELAHQMFCRYAFGKNSPPHGFNELADEAAKIAGNRPKALKYVGSSFRRLDKEQWVKMLSEFRSNGNKLKISYDELDGKGQDYVACLTNGSNSQVKAEWIHLALGVSILLNIRSDGTTILKHLSYNRSMAQQAKIWWYENLERVCKKYNICGIDSSTDGGGSTYGQCSNSQFQRNMDASPGGNKTSNQSTKDSPRASQVEKEKIEYCEPHVYITPAIFSDGTRAPKYVESSRRVTQVHHAKTWWPENCEKVYENHNNIYGIDRSIDGGDKFEGKSKVSDGGLDGKDQGSMYGQSSNSELQINMDADNRRCEPVSEMLFKNYNVCSPNGLTDVNCSNPQSQRKLDASLKKDKIVHEWIRTGSGFFFDFQGPKSIVSAAQVDEKNFEYCEQGVYITLGILSGGIIVLKHLEFSRRMAQQAKVWWSENWIKVYQEHNICGIDKSFDGRFDDRRVIRQLRPN

>L6[Linum usitatissimum] AAA91022.1

MSYLREVATAVALLLPFILLNKFWRPNSKDSIVNDDDDSTSEVDAISDSTNPSGSFPSVEYEVFLSFRGPDTREQFTDFLYQSLRRYKIHTFRDDDELLKGKEIGPNLLRAIDQSKIYVPIISSGYADSKWCLMELAEIVRRQEEDPRRIILPIFYMVDPSDVRHQTGCYKKAFRKHANKFDGQTIQNWKDALKKVGDLKGWHIGKNDKQGAIADKVSADIWSHISKENLILETDELVGIDDHITAVLEKLSLDSENVTMVGLYGMGGIGKTTTAKAVYNKISSCFDCCCFIDNIRETQEKDGVVVLQKKLVSEILRIDSGSVGFNNDSGGRKTIKERVSRFKILVVLDDVDEKFKFEDMLGSPKDFISQSRFIITSRSMRVLGTLNENQCKLYEVGSMSKPRSLELFSKHAFKKNTPPSYYETLANDVVDTTAGLPLTLKVIGSLLFKQEIAVWEDTLEQLRRTLNLDEVYDRLKISYDALNPEAKEIFLDIACFFIGQNKEEPYYMWTDCNFYPASNIIFLIQRCMIQVGDDDEFKMHDQLRDMGREIVRREDVLPWKRSRIWSAEEGIDLLLNKKGSSKVKAISIPWGVKYEFKSECFLNLSELRYLHAREAMLTGDFNNLLPNLKWLELPFYKHGEDDPPLTNYTMKNLIIVILEHSHITADDWGGWRHMMKMAERLKVVRLASNYSLYGRRVRLSDCWRFPKSIEVLSMTAIEMDEVDIGELKKLKTLVLKFCPIQKISGGTFGMLKGLRELCLEFNWGTNLREVVADIGQLSSLKVLKTTGAKEVEINEFPLGLKELSTSSRIPNLSQLLDLEVLKVYDCKDGFDMPPASPSEDESSVWWKVSKLKSLQLEKTRINVNVVDDASSGGHLPRYLLPTSLTYLKIYQCTEPTWLPGIENLENLTSLEVNDIFQTLGGDLDGLQGLRSLEILRIRKVNGLARIKGLKDLLCSSTCKLRKFYITECPDLIELLPCELGGQTVVVPSMAELTIRDCPRLEVGPMIRSLPKFPMLKKLDLAVANITKEEDLDAIGSLEELVSLELELDDTSSGIERIVSSSKLQKLTTLVVKVPSLREIEGLEELKSLQDLYLEGCTSLGRLPLEKLKELDIGGCPDLTELVQTVVAVPSLRGLTIRDCPRLEVGPMIQSLPKFPMLNELTLSMVNITKEDELEVLGSLEELDSLELTLDDTCSSIERISFLSKLQKLTTLIVEVPSLREIEGLAELKSLRILYLEGCTSLERLWPDQQQLGSLKNLNVLDIQGCKSLSVDHLSALKTTLPPRARITWPDQPYR

>L[Linum usitatissimum] AAD25969.1

MSYLREVATAVALLLPFILLNKFWRPNSKDSIVNDDDDSTSEVDAIPDSTNPSGSFPSVEYEVFLSFRGPDTREQFTDFLYQFLRRYKIHTFRDDDELRKGKEIGPNLLRAIDQSKIYVPIISSGYADSKWCLMELAEIVRRQEEDPRRIILPIFYMVDPSDVRHQTGCYKKAFRKHANKFDGQTIQNWKDALKKVGDLKGWHIGKNDKQGAIADEVLADIWSHISKENLILETDELVGIDDHITAVLEKMSLDSENVTMVGLYGMGGIGKTTTAKAVYNKISSCFDRCCFIDNIRETQDQKDGVVVLQKKLVSEILRIDSGSVGFNNDSGGRKMIKERVSRFKILVVLDDVDEKFKFEDMLGSPKDFISQSRFIITSRSMRVLGTLNENQCKLYEVGSMSKPRSLELFSKHAFKKNTPPSDYEILANDVVDTTAGLPLTLKVIGSLLFKQKIGVWEDTLEQLRKTLNLDEVYDRLKISYDALKPEAKEIFLDIACFFIGEKKEEPYYMWTDCNFYPASNITFLIQRCMIQVGNNDEFKMHDQLRDMGREIVRREDVRPWKRSRIWSAEEGIDLLLNKKGSSKVKAISIICGADYEFKSECFLNLSELRYLYATFAMLTGDFNNLLPNLKWLELPVYDHGEDDPPLTNFTMKNLIIVILEYSRITADDWGGWRNMMKMPERLKVVRLSSNYSSSGRLFRLSGCWRFPKSIEILSMTEIEMDEVDIGELKKLKTLVLGLCKIQKISGGTFGMLKGLIELDLLSLKCTNLREVVADIGQLSSLKVLKTLEVEEVEIKEFPSGLKELSTSSRIPNLSQLLDLEVLVVYDCKDGIDMPPASPSEDESSVWWKVSKLKSLQLEKTRINVNVVDDASSGGHLPRYLLPTSLTSLKIDRCTEPTWLPGIENLENLTSLEVNDIFQTLGGDLDGLQGLRSLEILRIRKVNGLARIKGLKDLLCSSTCKLRKLYIRECPDLIELLPCELGGQTVVVPSMAELTISDCPRLEVGPMIRSLPKFPMLKKLDLAVANITKEEDLDAIGSLEELVRLELVLDDTCSGIERIASLSKLQKLTTLVVKVPSLREIEGLAELKSLQRLILVGCTSLGRLPLEKLKELDIGGCPDLAELVQTVVAVPSLVELTIRDCPRLEVGPMIQSLPKFPMLNKLTLSMVNITKEDELAVLGSLEELDSLVLKLDDTCSGIERISFLSKLQKLTTLVVEVPSLREIEGLAELKSLQRLILVGCTSLGRLPLEKLKELDIGGCPDLAELVQTVVAVPSLVELTIRDCPRLEVGPMIQSLPKFPMLNKLMLSMVNITKEDELAVLGSLEELDSLVLKLDDTCSGIERISFLSKLQKLTTLVVEVPSLREIEGLAELKSLQRLTLEGCTSLGRLRLEKLKELDIGGCPDLTELVQTVVAVPSLVELTIRDCPRLEVGPMIQSLPNFPMLNELTLSMVNITKEDELEVLGSLEELRSLWLKLDDTCSSIERISSLSKLQKLTRLKVEVPSLREIEGLAELKSLQSLYLQGCTSLERLWPDQQQLGSLKNLIVINIRGCKSLSVDHLSALNTTLPPDVIIIWPDQPYSRW

>M[Linum usitatissimum] AAB47618.1

MSYLRDVATAVALLLDNLCCGRPNLNNDNEDTIQQTDSTSPVVDPSSSSQSMDSTSVVDAISDSTNPSASFPSVEYDVFLSFRGPDTRYQITDILYRFLCRSKIHTFKDDDELHKGEEIKVNLLRAIDQSKIYVPIISRGYADSKWCLMELAKIVRHQKLDTRQIIIPIFYMVDPKDVRHQTGPYRKAFQKHSTRYDEMTIRSWKNALNEVGALKGWHVKNNDEQGAIADEVSANIWSHISKENFILETDELVGIDDHVEVILEMLSLDSKSVTMVGLYGMGGIGKTTTAKAVYNKISSHFDRCCFVDNVRAMQEQKDGIFILQKKLVSEILRMDSVGFTNDSGGRKMIKERVSKSKILVVLDDVDEKFKFEDILGCPKDFDSGTRFIITSRNQNVLSRLNENQCKLYEVGSMSEQHSLELFSKHAFKKNTPPSDYETLANDIVSTTGGLPLTLKVTGSFLFRQEIGVWEDTLEQLRKTLDLDEVYDRLKISYDALKAEAKEIFLDIACFFIGRNKEMPYYMWSECKFYPKSNIIFLIQRCMIQVGDDGVLEMHDQLRDMGREIVRREDVQRPWKRSRIWSREEGIDLLLNKKGSSQVKAISIPNNMLYAWESGVKYEFKSECFLNLSELRLFFVGSTTLLTGDFNNLLPNLKWLDLPRYAHGLYDPPVTNFTMKKLVILVSTNSKTEWSHMIKMAPRLKVVRLYSDYGVSQRLSFCWRFPKSIEVLSMSGIEIKEVDIGELKNLKTLDLTSCRIQKISGGTFGMLKGLIELRLDSIKCTNLREVVADIGQLSSLKVLKTEGAQEVQFEFPLALKELSTSSRIPNLSQLLDLEVLKVYGCNDGFDIPPAKSTEDEGSVWWKASKLKSLKLYRTRININVVDASSGGRYLLPSSLTSLEIYWCKEPTWLPGIENLENLTSLVVDDVDIFQTLGGDLDGLQGLRSLETLTITEVNGLTRIKGLMDLLCSSTCKLEKLEIKACHDLTEILPCELHDQTVVVPSFEKLTIRDCPRLEVGPMIRSLPKFPMLKKLDLAVANITKEEDLDVIGSLQELVDLRIELDDTSSGIERIASLSKLKKLTTLRVKVPSLREIEELAALKSLQRLILEGCTSLERLRLEKLKEPDIGGCPDLTELVQTVVVCPSLVELTIRDCPRLEVGPMIRSLPKFPMLKKLDLAVANIIEEDLDVIGSLEELVILSLKLDDTSSSSIERISFLSKLQKLFRLRVKVSSLREIEGLAELKSLQLLFLKGCTSLERLWPDEQQLDNNKSMRIDIRGCKSLSVDHLSALKSTLPPNVKIRWPDEKYK

>N[Nicotiana glutinosa] AAA50763.1

MASSSSSSRWSYDVFLSFRGEDTRKTFTSHLYEVLNDKGIKTFQDDKRLEYGATIPGELCKAIEESQFAIVVFSENYATSRWCLNELVKIMECKTRFKQTVIPIFYDVDPSHVRNQKESFAKAFEEHETKYKDDVEGIQRWRIALNEAANLKGSCDNRDKTDADCIRQIVDQISSKLCKISLSYLQNIVGIDTHLEKIESLLEIGINGVRIMGIWGMGGVGKTTIARAIFDTLLGRMDSSYQFDGACFLKDIKENKRGMHSLQNALLSELLREKANYNNEEDGKHQMASRLRSKKVLIVLDDIDNKDHYLEYLAGDLDWFGNGSRIIITTRDKHLIEKNDIIYEVTALPDHESIQLFKQHAFGKEVPNENFEKLSLEVVNYAKGLPLALKVWGSLLHNLRLTEWKSAIEHMKNNSYSGIIDKLKISYDGLEPKQQEMFLDIACFLRGEEKDYILQILESCHIGAEYGLRILIDKSLVFISEYNQVQMHDLIQDMGKYIVNFQKDPGERSRLWLAKEVEEVMSNNTGTMAMEAIWVSSYSSTLRFSNQAVKNMKRLRVFNMGRSSTHYAIDYLPNNLRCFVCTNYPWESFPSTFELKMLVHLQLRHNSLRHLWTETKHLPSLRRIDLSWSKRLTRTPDFTGMPNLEYVNLYQCSNLEEVHHSLGCCSKVIGLYLNDCKSLKRFPCVNVESLEYLGLRSCDSLEKLPEIYGRMKPEIQIHMQGSGIRELPSSIFQYKTHVTKLLLWNMKNLVALPSSICRLKSLVSLSVSGCSKLESLPEEIGDLDNLRVFDASDTLILRPPSSIIRLNKLIILMFRGFKDGVHFEFPPVAEGLHSLEYLNLSYCNLIDGGLPEEIGSLSSLKKLDLSRNNFEHLPSSIAQLGALQSLDLKDCQRLTQLPELPPELNELHVDCHMALKFIHYLVTKRKKLHRVKLDDAHNDTMYNLFAYTMFQNISSMRHDISASDSLSLTVFTGQPYPEKIPSWFHHQGWDSSVSVNLPENWYIPDKFLGFAVCYSRSLIDTTAHLIPVCDDKMSRMTQKLALSECDTESSNYSEWDIHFFFVPFAGLWDTSKANGKTPNDYGIIRLSFSGEEKMYGLRLLYKEGPEVNALLQMRENSNEPTEHSTGIRRTQYNNRTSFYELING

>L5[Linum usitatissimum] AAD25972.1

MSYLREVATAVALLLPFILLNKFWRPNSKDSIVNDDDDSTSEVDAIPDSTNPSGSFPSVEYEVFLSFRGPDTREQFTDFLYHFLCRYKIHTFRDDDELRKGEEIGPNLLRAIDQSKIYVPIISSGYADSKWCLMELAEIVRRQEEDPRRIILPIFYMVDPSDVRHQTGCYKKAFRKHANKFDGQTIQNWKDALKKVGDLKGWHIGKNDKQGAIADEVLADIWSHISKENLILETDELVGIDDHITAVLEKLSLDSENVTMVGLYGMGGIGKTTTAKAVYNKISSCFDRCCFIDNIRETQDQKDGVVVLQKKLVYEILRIDSGSVGFNNDSGGRKMIKERVSRFKILVVLDDVDEKFKFEDMLGSPKDFISQSRFIITSRSMRVLGTLNENQCKLYEVGSMSKPRSLELFSKHAFKKNTPPSDYETLANDVVDTTAGLPLTLKVIGSILFKQEIGVWKDTLQQLRKTLNLDEVYDRLKISYDALKPEAKEIFLDIACFFIGKNKEVPYYMWTDCNFYPASNIIFLIQRCMIQVGDDDKFKMHDQLRDMGREIVRREDIRPWKRSRIWSREEGIDLLLNKKGSSIVKAISMVPPWVSWDNNVKYEFKSECFLNLSELRYFYADPTILLTGDFNNLLPNLKWLELPFYKHGEDDPPLTNFTLKNLIIVILEHSSITADDWGGWSHMMKMAERLKVVRLSSNYSSSGRLFRHSGCWRFPKSIEVLSMFGMKMEEVDIGELKKLKTLHLSFCEIQKISGGTFGMLKGLRELCLGNKLVGTNLREVVADIGQLSSLKVLETTGAKEVEINEFPLGLKKLSTSSRIPNLSQLLDLEVLVVYDCKDGFDMPPASPSEDESSVWWKVSKLKSLRLENTRINVNVVDDASSGGHLPRYLLPTSLTYLKIYQCTEPTWLPGIENLENLTSLEVNDIFQTLGGDLDGLQGLRSLEILRIRKVNGLARIKGLKDLLCSSTCKLRKLKIRDCPDLIELLPCELGGQTVVVPSMAELTIRDCPRLEVGPMIRSLPKFPMLKNLDLAVANITKEEDLDAIGSLEELVSLELKLDDTSSGIERIVSLSKLQKLTTLVVKVPSLREIEGLAELKSLQRLTLEGCTSLGRLRLEKLKELDIGGCPDLTELVQTVVAVPSLVELTIRDCPRLEVGPMIQSLPNFPMLNELTLSMVNITKEDELEVLGSLEELRSLELKLDDTCSSIERISSLSKLQKLTTLEVEVPSLREIEGLAELKSLYELYLQGCTSLERLWPDQQQLGSLENLNEINIRGCKSLSVDHLSALKTTPPPRARITWPDQPYR

>RPM1[Arabidopsis thaliana] AGC12590.1

MASATVDFGIGRILSVLENETLLLSGVHGEIDKMKKELLIMKSFLEDTHKHGGNGSTTTTTQLFQTFVANTRDLAYQIEDILDEFGYHIHGYRSCAKIWRAFHFPRYMWARHSIAQKLGMVNVMIQSISDSMKRYYHSENYQAALLPPIDDGDAKWVNNISESSLFFSENSLVGIDAPKGKLIGRLLSPEPQRIVVAVVGMGGSGKTTLSANIFKSQSVRRHFESYAWVTISKSYVIEDVFRTMIKEFYKEADTQIPAELYSLGYRELVEKLVEYLQSKRYIVVLDDVWTTGLWREISIALPDGIYGSRVMMTTRDMNVASFPYGIGSTKHEIELLKEDEAWVLFSNKAFPASLEQCRTQNLEPIARKLVERCQGLPLAIASLGSMMSTKKFESEWKKVYSTLNWELNNNHELKIVRSIMFLSFNDLPYPLKRCFLYCSLFPVNYRMKRKRLIRMWMAQRFVEPIRGVKAEEVADSYLNELVYRNMLQVILWNPFGRPKAFKMHDVIWEIALSVSKLERFCDVYNDDSDGDDAAETMENYGSRHLCIQKEMTPDSIRATNLHSLLVCSSAKHKMELLPSLNLLRALDLEDSSISKLPDCLVTMFNLKYLNLSKTQVKELPKNFHKLVNLETLNTKHSKIEELPLGMWKLKKLRYLITFRRNDGHDSNWNYVLGTRVVPKIWQLKDLQVMDCFNAEDEIIKNLGCMTQLTRISLVMVRREHGRDLCDSLNKIKRIRFLSLTSIDEEEPLEIDDLIATASIEKLFLAGKLERVPSWFNTLQNLTYLGLRGSQLQENAILSIQTLPRLVWLSFYNAYMGPRLRFAQGFQNLKILEIVQMKHLTEVVIEDGAMFELQKLYVRACRGLEYVPRGIENLINLQELHLIHVSNQLVERIRGEGSVDRSRVKHIPAIKHYFRTDNGSFYVSLSS

>RPP13[Arabidopsis thaliana] AAF42831.1

MVDAITEFVVGKIGNYLIEEASMFMAVKEDLEELKTELTCIHGYLKDVEAREREDEVSKEWSKLVLDFAYDVEDVLDTYHLKLEERSQRRGLRRLTNKIGRKMDAYSIVDDIRILKRRILDITRKRETYGIGGLKEPQGGGNTSSLRVRQLRRARSVDQEEVVVGLEDDAKILLEKLLDYEEKNRFIISIFGMGGLGKTALARKLYNSRDVKERFEYRAWTYVSQEYKTGDILMRIIRSLGMTSGEELEKIRKFADEELEVYLHGLLEGKKYLVVVDDIWEREAWESLKRALPCNHEGSRVIITTRIKAVAEGVDGRFYAHKLRFLTFEESWELFEQRAFRNIQRNDEDLLKTGKEMVQKCRGLPLCIVVLAGLLSRKTPSEWNDVCNSLWRRLKDDSIHVAPIVFDLSFKELRHESKLCFLYLSIFPEDYEIDVEKLIRLLVAEGFIQGDEEMMMEDVARYYIEELIDRSLLEAVRRERGKVMSCRIHDLLRDVAIKKSKELNFVNVYNDHVAQHSSTTCRREVVHHQVKRYSSEKRKNKRMRSFLNFGLDNLVGPDFETTKLLRVLDVRRLGFPLKINGDLIPLRYLGIDDYSFSDRAAIISKLRFLQTLEVSTYSEYPIYDTIDLRKLTSLRHVIGQFVGELLIGDAANLQTLRFISSDSWNKLKPELLINLRDLEIYEDYDEDFDRRVSVSWASLTKLRSLRVLKLYYLRLESEEAVRSTDVISLSLESVTLEGITFEEDTMPFLQKMPRLEDLILLHCNYSGGKMSVSEQGFGRLRKLQIFIHNSLDELQIEEEAMPNLIELKITFKEVKKLIIPNRLRACMSYES

>Prf[Solanum lycopersicum] AAF76312.1

MAKECRDAIGTINLVKGQHLDRRTTNQLEDAIKHLTHVAVFLTNLEKRHPANGISVHLRPLFLEAHDGFSLMCSHPPRSQFTVKLDNIAEKFKSAKASRSTRQVIPELLQIIEPENIAKRIKASKPSRSSSPITVDMVGFIESLLGSVHRALFFISAGPPVSMLDKKLRHLQVFFRLISKRGIEHESMKDLFYHVEDVAYTAAQLCVLGSSCHMDNEFSKLLERISRPFSPGLRLVYLNALIGLNSSRSKTTMNAKYMLDFVSALQDDLRLRCDNRIRWLQRGLSYLCRFLRDIESYPVSHRQLISLQLNMEDLAIGSANAIYSYDEDMDKTSEIDHELFHLQMKFNYVKVEVDLIRLQNIQGTIIVPMKDLIDYVWEELMFFRSYFMDAFDQCKEQTRITVILNYIQSAVSQAWSVCDSLCHDLNQNDLAREINCLHFQLLLKFKFIKVAIRQMCPSISASSTPDHPMIDLLNFLPMNFEAIDSYSSMLKASFPSSSHRPNKDAESPNTSFLCGPNTDVYSFYSSSSRIPKMDEILKRFHEYILVNLLRKDETNLTFTIADEVKKFYDGLLLMVTYLIEPPVPHTECRKQNDLSMRHEAVAIEAESAVCLHYEDNMNNNRCREINQVLQFLTVTFWLIKSEGNLMDLLKHKSTLGNQVLDLIESAHEELILLRSILMDLLRKKLYRLDDLLMHAEVTAKRLAIFSGSCYEYFMNGSSTEKMRPLLSDFLQEIESVKVEFRNVCLQVLDISPFSLTDGEGLVNFLLKNQAKVPNDDAVSSDGSLEDASSTEKMGLPSDFLREIESVEIKEARKLYDQVLDATHCETSKTDGKSFINIMLTQQDKVLDYDAGSVSYLLNQISVVKDKLLHIGSLLVDIVQYRNMHIELTDLAERVQDKNYICFFSVKGYIPAWYYTLYLSDVKQLLKFVEAEVKIICLKVPDSSSYSFPKTNGLGYLNCFLGKLEELLRSKLDLIIDLKHQIESVKEGLLCLRSFIDHFSESYDEHDEACGLIARVSVMAYKAEYVIDSCLAYSHPLWYKVLWISEVLENIKLVNKVVGETCERRNTEVTVHEVAKTTTNVAPSFSAYTQRANEEMEGFQDTIDELKDKLLGGSPELDVISIVGMPGLGKTTLAKKIYNDPEVTSRFDVHAQCVVTQLYSWRELLLTILNDVLEPSDRNEKEDGEIADELRRFLLTKRFLILIDDVWDYKVWDNLCMCFSDVSNRSRIILTTRLNDVAEYVKCESDPHHLRLFRDDESWTLLQKEVFQGESCPPELEDVGFEISKSCRGLPLSVVLVAGVLKQKKKTLDSWKVVEQSLSSQRIGSLEESISIIGFSYKNLPHYLKPCFLYFGGFLQGKDIHVSKMTKLWVAEGFVQANNEKGQEDTAQGFLDDLIGRNLVMAMEKRPNAKVKTCRIHDLLHKFCMEKAKQEDFLLQINSGEGVFPERLEEYRLFVHSYQDEIDLWRPSRSNVRSLLFNAIDPDNLLWPRDISFIFESFKLVKVLDLESFNIGGTFPTEIQYLIQMKYFAAQTDANSIPSSIAKLENLETFVVRGLGGEMILPCSLLKMVKLRHIHVNDRVSFGLRENMDVLTGNSQLPNLETFSTPRLFYGKDAEKILRKMPKLRKLSCIFSGTFGYSRKLKGRCVRFPRLDFLSHLESLKLVSNSYPAKLPHKFNFPSQLRELTLSKFRLPWTQISIIAELPNLVILKLLLRAFEGDHWEVKDSEFLELKYLKLDNLKVVQWSISDDAFPKLEHLVLTKCKHLEKIPSRFEDAVCLNRVEVNWCNWNVANSAQDIQTMQHEVIANDSFTVTIQPPDWSKEQPLDS

>Mi1[Solanum lycopersicum] AAC97933.1

MEKRKDIEEANNSLVLFSALSKDIANVLIFLENEENQKALDKDQVEKLKLKMAFICTYVQLSYSDFEQFEDIMTRNRQEVENLLQSLLDDDVLTSLTSNMDDCISLYHRSYKSDAIMMDEQLDFLLLNLYHLSKHHAEKIFPGVTQYEVLQNVCGNIRDFHGLILNGCIKHEMVENVLPLFQLMAERVGHFLWEDQTDEDSRLSELDEDEHNDRDSRLFQLTHLLLKIVPTELEVMHICYTNLKASTSAEVGRFIKKLLETSPDILREYIIQLQEHMLTVIPPSTLGARNIHVMMEFLLLILSDMPKDFIHHDKLFDLLAHVGTLTREVSTLVRDLEEKLRNKEGNNQTNCATLDLLENIELLKKDLKHVYLKAPNSSQCCFPMSDGPLFMHLLHMHLNDLLDSNAYSISLIKEEIELVSQELEFIRSFFGDAAEQGLYKDIWARVLDVAYEAKDVIDSIIVRDNGLLHLIFSLPITIKKIKLIKEEISALDENIPKDRGLIVVNSPKKPVERKSLTTDKIIVGFEEETNLILRKLTSGPADLDVISITGMPGSGKTTLAYKVYNDKSVSRHFDLRAWCTVDQGYDDKKLLDTIFSQVSGSDSNLSENIDVADKLRKQLFGKRYLIVLDDVWDTTTLDELTRPFPEAKKGSRIILTTREKEVALHGKLNTDPLDLRLLRPDESWELLEKRTFGNESCPDELLDVGKEIAENCKGLPLVADLIAGVIAGREKKRSVWLEVQSSLSSFILNSEVEVMKVIELSYDHLPHHLKPCLLHFASWPKDTPLTIYLLTVYLGAEGFVEKTEMKGIEEVVKIYMDDLISSSLVICFNEIGDILNFQIHDLVHDFCLIKARKENLFDRIRSSAPSDLLPRQITIDYDEEEEHFGLNFVMFDSNKKRHSGKHLYSLRINGDQLDDSVSDAFHLRHLRLIRVLDLEPSLIMVNDSLLNEICMLNHLRYLRIRTQVKYLPFSFSNLWNLESLFVSNKGSILVLLPRILDLVKLRVLSVGACSFFDMDADESILIAKDTKLENLRILGELLISYSKDTMNIFKRFPNLQVLQFELKESWDYSTEQHWFPKLDCLTELETLCVGFKSSNTNHCGSSVATNRPWDFHFPSNLKELLLYDFPLTSDSLSTIARLPNLENLSLYDTIIQGEEWNMGEEDTFENLKFLNLRLLTLSKWEVGEESFPNLEKLKLQECGKLEEIPPSFGDIYSLKFIKIVKSPQLEDSALKIKKYAEDMRGGNELQILGQKNIPLFK

>Mla1[Hordeum vulgare subsp. vulgare] AAG37356.1

MDIVTGAISNLIPKLGELLTEEFKLHKGVKKNIEDLGKELESMNAALIKIGEVPREQLDSQDKLWADEVRELSYVIEDVVDKFLVQVDGIQFDDNNNKFKGFMKRTTELLKKVKHKHGIAHAIKDIQEQLQKVADRRDRNKVFVPHPTRTIAIDPCLRALYAEATELVGIYGKRDQDLMRLLSMEGDDASNKRLKKVSIVGFGGLGKTTLARAVYEKIKGDFDCRAFVPVGQNPHMKKVLRDILIDLGNPHSDLAMLDANQLIKKLREFLENKRYLVIIDDIWDEKLWEGINFAFSNRNNLGSRLITTTRIVSVSNSCCSSHGDSVYQMEPLSVDDSRILFWKRIFPDENGCLNEFEQVSRDILKKCGGVPLAIITIASALAGDQKMKPKCEWDILLQSLGSGLTEDNSLEEMRRILSFSYSNLPSHLKTCLLYLCIYPEDSKIHRDELIWKWVAEGFVHHENQGNSLYLLGLNYFNQLINRSMIQPIYGFNDEVYVCRVHDMVLDLICNLSREAKFVNLLDGSGNSMSSQGNCRRLSLQKRNEDHQAKPITDIKSMSRVRSITIFPPAIEVMPSLSRFDVLRVLDLSRCNLGENSSLQLNLKDVGHLTHLRYLGLEGTNISKLPAEIGKLQFLEVLDLGNNHNLKELPSTVCNFRRLIYLNLFGCPVVPPVGVLQNLTSIEVLRGILVSVNIIAQELGNLERLRVLDICFRDGSLDLYKDFVKSLCNLHHIESLRIECNSRETSSFELVDLLGERWVPPVHFREFVSSMPSQLSALRGWIKRDPSHLSNLSELILSSVKDVQQDDVEIIGGLLCLRRLFIITSTDQTQRLLVIRADGFRCTVDFRLDCGSATQILFEPGALPRAVRVWFSLGVRVTKEDGNRGFDLGLQGNLFSLREFVSVYMYCGGARVGEAKEAEAAVRRALEAHPSHPRIYIQMRPHIAKGAHDDDLCEDEEEN

>Mla6[Hordeum vulgare subsp. vulgare] CAC29242.1

MDIVTGAISNLIPKLGELLTEEFKLHKGVKKNIEDLGKELESMNAALIKIGEVPREQLDSQDKLWADEVRELSYVIEDVVDKFLVQVDGIQSDDNNNKFKGLMKRTTELLKKVKHKHGIAHAIKDIQEQLQKVADRRDRNKVFVPHPTRPIAIDPCLRALYAEATELVGIYGKRDQDLMRLLSMEGDDASNKRLKKVSIVGFGGLGKTTLARAVYEKIKGDFDCRAFVPVGQNPDMKKVLRDILIDLGNPHSDLAMLDANQLIKKLHEFLENKRYLVIIDDIWDEKLWEGINFAFSNRNNLGSRLITTTRIVSVSNSCCSSDGDSVYQMEPLSVDDSRMLFSKRIFPDENGCINEFEQVSRDILKKCGGVPLAIITIASALAGDQKMKPKCEWDILLRSLGSGLTEDNSLEEMRRILSFSYSNLPSHLKTCLLYLCVYPEDSMISRDKLIWKWVAEGFVHHENQGNSLYLLGLNYFNQLINRSMIQPIYNYSGEAYACRVHDMVLDLICNLSYEAKFVNLLDGTGNSMSSQSNCRRLSLQKRNEDHQVRPFTDIKSMSRVRSITIFPSAIEVMPSLSRFDVLRVLDLSRCNLGENSSLQLNLKDVGHLTHLRYLGLEGTNISKLPAEIGKLQFLEVLDLGNNRNIKELPSTVCNFRRLIYLNLVGCQVVPPVGLLQNLTAIEVLRGILVSLNIIAQELGKLKSMRELEIRFNDGSLDLYEGFVKSLCNLHHIESLIIGCNSRETSSFEVMDLLGERWVPPVHLREFESSMPSQLSALRGWIKRDPSHLSNLSDLVLPVKEVQQDDVEIIGGLLALRRLWIKSNHQTQRLLVIPVDGFHCIVDFQLDCGSATQILFEPGALPRAESVVISLGVRVAKEDGNRGFDLGLQGNLLSLRRHVFVLIYCGGARVGEAKEAKAALRRAQEAHPDHLRIYIDMRPCIAEGAHDDDLCEGEEEN

>Mla12[Hordeum vulgare subsp. vulgare] AAO43441.1

MDIVTGAISNLIPKLGELLTEEFKLHKGVKKNIEDLGKELESMNAALIKIGEVPREQLDSQDKLWADEVRELSYVIEDVVDKFLVQVDGIKSDDNNNKFKGLMKRTTELLKKVKHKHGIAHAIKDIQEQLQKVADRRDRNKVFVPHPTRTIAIDPCLRALYAEATELVGIYGKRDQGLMRLLSMEGDDASNKRLKKVSIVGFGGLGKTTLARAVYEKIKGDFDCRAFVPVGQNPDMKKVLRDILIDLGNPHSDLAMLDANQLIKKLHEFLENKRYLVIIDDIWDEKLWEGINFAFSNRNNLGSRLITTTRIVSVSNSCCSSDGDSVYQMEPLSVDDSRMLFYKRIFPDENACINEFEQVSRDILKKCGGVPLAIITIASALAGDQKMKPKCEWDILLRSLGSGLTEDNSLEEMRRILSFSYSNLPSNLKTCLLYLCVYPEDSMISRDKLIWKWVAEGFVHHENQGNSLYLLGLNYFNQLINRSMIQPIYNYSGEAYACRVHDMVLDLICNLSREAKFVNLLDGTGNSMSSQSNCRRLSLQKRNEDHQARPLIDIKSMSRVRSITIFPPAIEVMPSLSRFEVLCVLDLSKCNLGEDSSLQLNLKDVGQLIQLRYLGLECTNISKLPTEIGKLQFLEVLDLGNNPNLKELPSTIRNFRRLIYLNLVGCQVIPPVGVLQNLTSIEVLRGILVYLNIIAQELGNLERVRDLEIRFNDGSLDLYEGLVNSLCNLHHIESLNIRCNPGETSSFELMDLLEERWVPPVHLREFKSFMPSQLSALRGWIQRDPSHLSNLSELTLWPVKDVQQDDVEIIGGLLSLRRLWIVKSIHQTQRLLVIRADGFRSMVEFRLDCGSATQILFEPGALPRAESVVISLGVRVAKEDGNRGFDLGLQEAKDVSLRWDVFVLLYCGGARVGEAKEAEAAVRRALEAHPRHPRIYIDMRPDIQEGAHDDDLCENEDEGEN

>Mla13[Hordeum vulgare subsp. vulgare] AAO16014.1

MDIVTGAISNLIPKLGELLTEEFKLHKGVKKNIEDLGKELESMNAALIKIGEVPREQLDSQDKLWADEVRELSYVIEDVVDKFLVQVDGIKSDDNNNKFKGLMKRTTELLKKVKHKHGIAHAIKDIQEQLQKVADRRDRNKVFVPHPTRTIAIDPCLRALYAEATELVGIYGKRDQGLMRLLSMEGDDASNKRLKKVSIVGFGGLGKTTLARAVYEKIKGDFDCRAFVPVGQNPDMKKVLRDILIDLGNPHSDLAMLDANQLIKKLHEFLENKRYLVIIDDIWDEKLWEGINFAFSNRNNLGSRLITTTRIVSVSNSCCSSDGDSVYQMEPLSVDDSRMLFYKRIFPDENACINEFEQVSRDILKKCGGVPLAIITIASALAGDQKMKPKCEWDILLRSLGSGLTEDNSLEEMRRILSFSYSNLPSNLKTCLLYLCVYPEDSMISRDKLIWKWVAEGFVHHENQGNSLYLLGLNYFNQLINRSMIQPIYNYSGEAYACRVHDMVLDLICNLSNEAKFVNLLDGTGNSMSSQSNCRRLSLQKRNEDHQARPFTDIKSMSRVRSITIFPSAIEVMPSLSRFDVLRVLDLSRCNLGENSSMQLNLKGVGHLTHLRYLGLEGTNISKLPAEIGKLQFLEVLDLENNHNLKELPSTVCNFRRLIYLNLVGCQVVPPVGVLQNLTSIEVLSGILVSLNIIAQELGNLKRLRELNILFNDGSLDLYEGFVKSLCNLHHIESLIIGCNSRETSSFELMDLLGERWVPPVHFREFVSSMPSQLSALRGWIKRDPSHLSNLSELILTSVKEVQQDDVVIIGALSSLRRLCIKSTYQTQRLLVIPADGFRCIVGFHLDCGSATQILFEPGALPRAESVVISLGVRVAKEDGNRGFDLGLQGNLLSLRRDVFVSIYCGGARVGEAKEAEAAVRRALDAHPSHPPIYFEMRPHIAKGAHDDDLCEERRRTDF

>BS2[Capsicum chacoense] AAF09256.1

MAHASVASLMRTIESLLTFNSPMQSLSCDHREELCALREKVSSLEVFVKNFEKNNVFGEMTDFEVEVREVASAAEYTIQLRLTGTVLGENKSQKKKARRRFRQSLQQVAEDMDHIWKESTKIQDKGKQVSKESLVHDFSSSTNDILKVKNNMVGRDDQRKQLLEDLTRSYSGEPKVIPIVGMGGIGKTTLAKEVYNDESILCRFDVHAWATISQQHNKKEILLGLLHSTIKMDDRVKMIGEAELADMLQKSLKRKRYLIVLDDIWSCEVWDGVRRCFPTEDNAGSRILLTTRNDEVACYAGVENFSLRMSFMDQDESWSLFKSAAFSSEALPYEFETVGKQIADECHGLPLTIVVVAGLLKSKRTIEDWKTVAKDVKSFVTNDPDERCSRVLGLSYDHLTSDLKTCLLHFGIFPEDSDIPVKNLMRSWMAEGFLKLENDLEGEVEKCLQELVDRCLVLVSKRSRDGTKIRSCKVHDLIYDLCVREVQRENIFIMNDIVLDVSYPECSYLCMYKMQPFKRVTGDEINYCPYGLYRALLTPVNRQLRDHDNNNLLKRTHSVFSFHLEPLYYVLKSEVVHFKLLKVLELRHRQIDGFPREILSLIWLRYLSLFSYGNFDVPPEICRLWNLQTFIVQRFRSDIIIFAEEIWELMQLRHLKLPRFYLPDCPSGSVDKGRHLDFSNLQTISYLSPRCCTKEVIMGIQNVKKLGISGNKDDYKSFRDSGLPNNLVYLQQLEILSLISVDYSLLPVIISSAKAFPATLKKLKLERTYLSWSYLDIIAELPNLEVLKLMDDACCGEEWHPIVMGFNRLKLLLIKYSFLKFWKATNDNFPVLERLMIRSCKNLKEIPIEFADIHTLQLIELRECPPKLGESAARIQKEQEDLGNNPVDVRISNPLKESDSDSEEH

>PM3b[Triticum aestivum] AAQ96158.1

MAERVVTMAIGPLVSMLKDKASSYLLDQYKVMEGMEEQHKILKRKLPAILDVITDVEEQAMAQREGAKAWLQELRTVAYVANEVFDEFKYEALRREAKKNGHYIKLGFDVIKLFPTHNRVAFRYKMGRKLCLILQAVEVLIAEMQVFGFKYQPQPPVSKEWRHTDYVSIDPQEIASRSRHEDKKNIIGILVDEASNADLTVVPVVAMGGLGKTTLAQLIYNDPEIQKHFQLLLWVCVSDTFDVNSLAKSIVEASPNKNVDTDKPPLARLQKLVSGQRYLLVLDDVWDNKELRKWERLKVCLQHGGMGSAVLTTTRDKRVAEIMGADRAAYNLNALEDHFIKEIIVDRAFSSENGKIPELLEMVGEIVKRCCGSPLAASALGSVLRTKTTVKEWNAIASRSSICTEETGILPILKLSYNDLPSHMKQCFAFCAVFPKDYKIDVAKLIQLWIANGFIPEHKEDSLETIGQLIFDELASRSFFLDIEKSKEDWEYYSRTTCKIHDLMHDIAMSVMEKECVVATMEPSEIEWLPDTARHLFLSCEETERILNDSMEERSPAIQTLLCDSNVFSPLKHLSKYSSLHALKLCIRGTESFLLKPKYLHHLRYLDLSESSIKALPEDISILYNLQVLDLSYCNYLDRLPRQMKYMTSLCHLYTHGCRNLKSMPPGLENLTKLQTLTVFVAGVPGPDCADVGELHGLNIGGRLELCQVENVEKAEAEVANLGGQLELQHLNLGDQLELRRVENVKKAEAKVANLGNKKDLRELTLRWTEVGDSKVLDKFEPHGGLQVLKIYKYGGKCMGMLQNMVEIHLSGCERLQVLFSCGTSFTFPKLKVLTLEHLLDFERWWEINEAQEEQIIFPLLEKLFIRHCGKLIALPEAPLLGEPSRGGNRLVCTPFSLLENLFIWYCGKLVPLREAPLVHESCSGGYRLVQSAFPALKVLALEDLGSFQKWDAAVEGEPILFPQLETLSVQKCPKLVDLPEAPKLSVLVIEDGKQEVFHFVDRYLSSLTNLTLRLEHRETTSEAECTSIVPVDSKEKWNQKSPLTVLELGCCNSFFGPGALEPWDYFVHLEKLEIDRCDVLVHWPENVFQSLVSLRTLLIRNCKNLTGYAQAPLEPLASERSQHPRGLESLCLRNCPSLVEMFNVPASLKKMTIGGCIKLESIFGKQQGMAELVQVSSSSEAIMPATVSELPSTPMNHFCPCLEDLCLSACGSLPAVLNLPPSLKTLEMDRCSSIQVLSCQLGGLQKPEATTSRSRSPIMPQPLAAATAPAAREHLLPPHLEYLTILNCAGMLGGTLRLPAPLKRLFIIGNSGLTSLECLSGEHPPSLESLWLERCSTLASLPNEPQVYRSLWSLEITGCPAIKKLPRCLQQQLGSIKRKWLDARYEVTEFKPLKPKTWKEIPRLVRERRQACRS

>LR10[Triticum aestivum] AAQ01784.1

MAPCLVSASTGAMGSLLTKLETMLDDEYILLNVRRDIKFVIHELAMWQSFLLDVADTEEPGQHDKSCADLVRELSYDIEDKIDNSMSLMLHHACPKSGIKKHMSKFKNLLPVKIPYQIAKDIKDIKSQILEVSNRCERYRFEDVCLARTEFVDPRLCTVDTCAADLVGIDGPKHELVKWLRNGEDESVHQQKVVSIVGCAGLGKTTLAKQVYDELRINFEYRAFVSISRSPNMATILKCVLSQFHAQDYSSDESEIPKLVDQIRDLLQDKRYFVIIDDIWDMKTWDVLKCALCKNSCGSVIMTTTRIYDVAKSCCSSNGDLVYNIQPLSVADSEELFLNRVFGHEKGFPPELKEVSKDVLRKCGGLPLAINAISSLLAAEKIEEWDRVGLSNVFAQGEKSDIDAMKYKLSLCYFDLPLHLRSCLLYLIMFPEDCLIEKERLVHRWISEGFIRNEDGEDLVEVGERYLYELVNRSLIESVGVPYDGKARFYRVHNVILDFLMIKSMEENFCTLTSNQSRLDYKVRRLSLFANKDPSCIAQLDLSHARSLGASGHLGQLISSVKSNALRVLDVQDCSELGNHHVKDIGRNPLLRYLNISGTDVTELPIQIGDMGFLETLDASFTELVEMPGSITRLRQLQRLFVSDETKLPDEIGNMKRLQELGDINAFKQSVNFLNELGKLTGLRKLGIIWDTNDILKSGKGSSKEKRLVSSLSKLDAGRLSNLYVTFYLREKDGFIGHPFLPALNSIREVYLRRGRMCWMNKWLLSLANLEKLYISGGDEIEQDDLRTVGSIPTLVEFKLYSGCLGPIIISSGFEQLERLELKFSFSQLTFEVGAMPNLKKLDLHVYLSKFKSAGAGFDFGIQHLSSLACVSIVIFCEGVSAAYVEAAEGAFKSMVNAHPNPNRPMLEMTRESADFMSQDE

>AhRRS5

MAESAIAFLLQRLVSVFENEVTWFPGIQEEVVHLKGHLGVIRAFLRVADAKQESDEELKVCIKQLRDIAHDAEDLLDELELVQAYDHTNGFSVILSRFSGQIRHMKARYRIASDLKGINSRMRTILGVLAKFDTASQASNYTGKAWHDQRGDALLLENTDLVGIEEPKKQLISWLIKGCPGRKVISVTGMGGMGKTTVVKKVYDDPEVIKHFKACVWVTVSQSFKTEELLRDLVQKIFSEIRRPVPDGLESMRSDKLKLIIKDMLQRRRYLVVFDDVWHMHEWEAVKYALPDNNCGSRVMITTRKSDLASACSIQSKGKVYNLQPLKEDEVWDLFTRKTFQGKSCPSYLTSICKCILRKCEGLPLAIVAISSVLAMKDKCRIEEWDMICHSLGAEIQDNDKLGNLKTVLGLSINDLPYYLKYCFLYLSIFPEDHLIERMRLIRLWIAEGFIEAKEGKTLEDVAEDYLKELLNRNLIQVAGTTTDGRVKTLRIHDLIREIIILKSKDENFATIVKEQSVPWPERLRRLSVHNTMPNGQQQRSVSQLRSLLMFGVAEQLSLCKLFPGGFRLLAVLDFQDAPLQKFPVAIGGLYCLRYLSLRNTKVNMVPGKILGKLKNLETLDLKKTSITELPADILNLKKLRHLLVYQVKVKGYGEFHSKLGFKAPSEIGYLQSLQKLCFVEANQGCGKIIRQLAELCQLRRLGIRNLREEDGKAFCLSIERLVNLCALSVTSEGENKVIALEFLSSPPPYLQRLYLSGRLLDLPDWMPSLHNLAKLFLKWSCLEQDPLEYLQDLPNLSHLELLQAYTGDTLHFQCGKFKKLKILGLDRFVELKQVILGKDAMPCLEKLIIQRCQLLKNVPSGVELLTKLKVLELFDMPDELMKTICPQGPGKDYWKVAHIPEVFSTYWRDGAWDVYPLESFKDCSPRSGTVMRSDERSTLSKV

>RPP8[Arabidopsis thaliana] BAC67706.1

MAESSVSFLLEKLTWLLQEEVNLQRGVREDVQYINDELERHKAILMAADSMEDKDPELKVWVKRVRVIAQDMEDAIDEYYLRLVDHQQGKIRSYFHKILFGIKTMKARHKIASNIQGIKSKVEVILRRRPIIPDVASSSSQRFSSRLDSQGDALLLEEADLVGIDQPKKQLTDLLFKDESKREVISIYGMGGLGKTTLAKQVYDDPKVKKRFRIHAWVNLSQSIKMEEILKDLVQKLHNVFGKPAPGSIGTMNNDDLKELIKNLLQRSRYLIVLDDVWNVKVWDDVKHSLPNNNRGSRVMLTTRKKDIVRAELGKDFHLAFLPEQEAWSLFCRKTFQGNSCPPHLEEVCRNILKLCGGLPLAIVAISGALATRGRTNIEEWQIVCRSFGSEIEGNDKLEDMKKVLSLSFNELPYHLKSCLLYLSIFPEFHAIEHMRLIRLLIAEGFVNSENGKTLEEVADRYLKELLNRSLLQVVEKTSDGRIKTCRMHDLLREIVNFKSRDQNFATVAKEQDMVWPERVRRLSVINSSHNVHKQNKTIFKLRSLLMFAISDSVNHFSIHELCSSTGVKLLNVLDLQDAPLEDFPVEIVNLYLLKHLSLKNTKVKSIPGSIKKLKYLETLDLKHTYVTELPVEVAELKRLRHLLVYRYEIESYAHFHSRHGFKVAAPIGNMLSLQKLCFIEVDQGSRALMVELGKLTQLRRLGIRKMRKEDGAALCSSIEKMINLRSLNITAIEDDEIIDIHNISKPPQYLQQLYLSGRLEKFPQWINSLKNLVKVFLKWSRLKEDPLVYLQDLPNLRHLEFLQVYVGDTLNFNAKGFPSLKVLGLDDLEGLKHMIIEEGAMQSLKKLVMQRCGSFKNVPLGIEHLTKLKTIEFFDMPDELIMALRPNVGADYWRVQNVPTVYSTYWRDGGWDVYSLETFGERESDSNHSSAKRTRELPTLWKV

>RXO1[Zea mays] AAX31149.1

MAEIAVLLVLKKIAIALAGETLSFAKPLLAKKSESVAALPDDMKLISNELELIRAFLKEIGRKGWKSEVIETWIGQVRRLAYDMEDTVDHFIYVVGTHDQMGSCWDYMKKIAKKPRRLVSLDEIASEIKKIKQELKQLSESRDRWTKPLDGGSGIPAGSYETEKEMYLPGHDYTISDEELAGIDENKQTLISSLKFEDPSLRIIAVWGMGGVGKSTLVNNVYKNEGSNFDCRAWVSISQSYRLEDIWKKMLTDLIGKDKIEFDLGTMDSAELREQLTKTLDKRQYLIILDDVWMANVFFKIKEVLVDNGLGSRVIITTRIEEVASLAKGSCKIKVEPLGVDDSWHVFCRKAFLKDENHICPPELRQCGINIVEKCDGLPLALVAIGSILSLRPKNVDEWKLFYDQLIWELHNNENLNRVEKIMNLSYKYLPDYLKNCFLYCAMFPEDYLIHRKRLIRLWIAEGFIEQKGACSLEDTAESYLKELIRRSMLHVAERNCFGRIKCIRMHDLVRELAIFQSKREGFSTTYGGNNEAVLVGSYSRRVAVLQCSKGIPSTIDPSRLRTLITFDTSRALSVWYSSISSKPKYLAVLDLSSLPIETIPNSIGELFNLRLLCLNKTKVKELPKSITKLQNLQTMSLENGELVKFPQGFSKLKKLRHLMVSRLQDVTFSGFKSWEAVEPFKGLWTLIELQTLYAITASEVLVAKLGNLSQLRRLIICDVRSNLCAQLCGSLSKLCQLSRLTIRACNEDEVLQLDHLTFPNPLQTLSLDGRLSEGTFKSPFFLNHGNGLLRLMLFYSQLSENPVPHLSELSNLTRLSLIKAYTGQELYFQAGWFLNLKELYLKNLSRLNQIDIQEGALASLERITMKHLPELREVPVGFRFLKSLKTIFFSDMHPEFESSFQKEM

>Pi9[Oryza sativa Indica Group] ABB88855.1

MAETVLSMARSLVGSAISKAASAAANETSLLLGVEKDIWYIKDELKTMQAFLRAAEVMKKKDELLKVWAEQIRDLSYDIEDSLDEFKVHIESQTLFRQLVKLRERHRIAIRIHNLKSRVEEVSSRNTRYNLVEPISSGTEDDMDSYAEDIRNQSARNVDEAELVGFSDSKKRLLEMIDTNANDGPAKVICVVGMGGLGKTALSRKIFESEEDIRKNFPCIAWITVSQSFHRIELLKDMIRQLLGPSSLDQLLQELQGKVVVQVHHLSEYLIEELKEKRYFVILDDLWILHDWNWINEIAFPKNNKKGSRIVITTRNVDLAEKCATASLVYHLDFLQMNDAITLLLRKTNKNHEDMESNKNMQKMVERIVNKCGRLPLAILTIGAVLATKHVSEWEKFYEQLPSELEINPSLEALRRMVTLGYNHLPSHLKPCFLYLSIFPEDFEIKRNRLVGRWIAEGFVRPKVGMTTKDVGESYFNELINRSMIQRSRVGIAGKIKTCRIHDIIRDITVSISRQENFVLLPMGDGSDLVQENTRHIAFHGSMSCKTGLDWSIIRSLAIFGDRPKSLAHAVCLDQLRMLRVLDLEDVTFLITQKDFDRIALLCHLKYLSIGYSSSIYSLPRSIGKLQGLQTLNMLRTYIAALPSEISKLQCLHTLRCSRKFVYDNFSLNHPMKCITNTICLPKVFTPLVSRDDRAKQIAELHMATKSCWSESFGVKVPKGIGKLRDLQVLEYVDIRRTSSRAIKELGHLSKLRKLGVITKGSTKEKCKILYAAIEKLSSLQSLYVNAALLSDIETLECLDSISSPPPLLRTLGLNGSLEEMPNWIEQLTHLKKIYLLRSKLKEGKTMLILGALPNLMVLYLYWNAYLGEKLVFKTGAFPNLRTLRIYELDQLREMRFEDGSSPLLEKIEISCCRLESGIIGIIHLPRLKEISLEYKSKVARLGQLEGEVNTHPNRPVLRMDSDRRDHDLGAEAEGSSIEVQTADPVPDAEGSVTVAVEATDPLPEQEGESSQSQVITLTTNDSEEIGTAQAG

>I2C-1[Solanum lycopersicum] AAB63274.1

MEIGLAIGGAFLSSALNVLFDRLAPNGDLLNMFRKHTDDVELFEKLGDILLSLQIVLSDAENKKASNQFVSQWLHKLQTAVDAAENLIEQVNYEALRLKVETSNQQVSDLNLCLSDDFFLNIKKKLEDTIKKLEVLEKQIGRLGLKEHFISTKQETRTPSTSLVDDSGIFGRKNEIENLVGRLLSMDTKRKNLAVVPIVGMGGMGKTTLAKAVYNDERVQKHFGLTAWFCVSEAYDAFRITKGLLQEIGSTDLKADDNLNQLQVKLKADDNLNQLQVKLKEKLNGKRFLVVLDDVWNDNYPEWDDLRNLFLQGDIGSKIIVTTRKESVALMMDSGAIYMGILSSEDSWALFKRHSLEHKDPKEHPEFEEVGKQIADKCKGLPLALKALAGMLRSKSEVDEWRNILRSEIWELPSCSNGILPALMLSYNDLPAHLKQCFAYCAIYPKDYQFRKEQVIHLWIANGLVHQFHSGNQYFIELRSRSLFEMASEPSERDVEEFLMHDLVNDLAQIASSNHCIRLEDNKGSHMLEQCRHMSYSIGQDGEFEKLKSLFKSEQLRTLLPIDIQFHYSKKLSKRVLHNILPTLRSLRALSLSHYQIEVLPNDLFIKLKLLRFLDLSETSITKLPDSIFVLYNLETLLLSSCEYLEELPLQMEKLINLRHLDISNTRRLKMPLHLSRLKSLQVLVGAKFLVGGWRMEYLGEAHNLYGSLSILELENVVDRREAVKAKMREKNHVEQLSLEWSESISADNSQTERDILDELRPHKNIKAVEITGYRGTNFPNWVADPLFVKLVHLYLRNCKDCYSLPALGQLPCLEFLSIRGMHGIRVVTEEFYGRLSSKKPFNSLVKLRFEDMPEWKQWHTLGIGEFPTLEKLSIKNCPELSLEIPIQFSSLKRLDICDCKSVTSFPFSILPTTLKRIKISGCPKLKLEAPVGEMFVEYLSVIDCGCVDDISPEFLPTARQLSIENCHNVTRFLIPTATESLHIRNCEKLSMACGGAAQLTSLNIWGCKKLKCLPELLPSLKELRLTYCPEIEGELPFNLQILDIRYCKKLVNGRKEWHLQRLTELWIKHDGSDEHIEHWELPSSIQRLFIFNLKTLSSQHLKSLTSLQFLRIVGNLSQFQSQGQLSSFSHLTSLQTLQIWNFLNLQSLPESALPSSLSHLIISNCPNLQSLPLKGMPSSLSTLSISKCPLLTPLLEFDKGEYWTEIAHIPTIQIDEECM

>hero[Solanum lycopersicum] CAD29728.1

MEKGEKPLLLFEEQKKRITNLIDDFLNGLKQIMNEEEEFIASKLDVIQKLRMDLRLLRTFVLFGNSTNLDDFYYRMNIHINKFNILTETLFCKDDLILEKYHMECVAPLLLKEIRNYLSLKNDYVATAIEMKKFEYLIRNLHDLPKYCYDLLQPLMSEYKILRQVCTHLRDFYQLECNKTTKTEFLYTRYQVTVDRVTQFCFDLWTGKYRNYRYEYAFSKCSSKITSLLIDIIPLELEVLHISTSNLIKESRSKELEGFVKQILKASPRILQKHLIHLQGRMVADSYSATQSINVMMEFLLIFLTDIPKRFIHRGKLNSMLAHVGLLTRKISILMEESSTMNEAEFSAPYLLHEIERMKGDIKQIILKAPESSQLCFPMDDGFLFMNLLLRHLNDLLTSNSYSVSLIKKEIEMVKQSLEFLTASFRQTLDESTSGVVKDCWMCALDVAYEAEHVINSILVRDKALSHLLFSLPDVIDKIKFIVAQVTGLQLEAKNGDDPLDAKSSYEPIELTSSSFVEVTVGHEEDEARIIGQLLDEHESKLDVISIVGMPGVGKTTLANKVYNNTLVASHFKIRAKCTVSQNFNKSKVLREILQQVTASETNRSEDDLAEKLRVALLDKRYLIVLDDVWDIATGEMLIACFPKVERGNRVILTSRSGEVGLKVKCRSDPVDLQVLTDEKSWELFEKRVFRDEGSCPAELLDIGHQIVEKCKGLPLALVLIAGVIVRGREGKEKEKEKDFWVKIQNNLDSFTSSNINSQIMNVMQSSYDHLPYQLKPLLLYFARLQKSERTPVSMLMQLWMAEGLVDHDIPSKCSLEEVTESYLDALISSSLIMVDHIRSVSNWTSVMMRACYVHDVVHDFCSVKAGKEKFFKLINSGDPFHASDFLHHRLTIHTDDKKCVLFNSNKCSAGSKHLISLEVSSSLDNFGYIFHTRHMRLVRVLQLDDIVLQHHLVEEIGSLFHLRFLKIWTRDVKAIPLSWLNLQNLETLLISEEFSTIVLLPRLFKLSKLKHVSIDQSSFFDKEEVEVEVEEEEEVEVEVEEEDEDKEEEDTDNIQSRILEGENSKLTTLSKVDISYSQGTNDALEKFQNLEHLDCTIIVPECPPKHGDWFPKFDVLNKLQSLTAVYKWSHYGYPIIEYHFPTSLKDLRLHSFPITPALLSVIAALPQLEILAIFYSDFMEDKWDASKDIYQSLKTLSLSYIKLSEWEVDRSETFPKLEELILERCYKLTEIPSAFEDIETLKIIHLTNIKRELGDSAIEIKKQIVEITGVDRLQVHLSGLYE

>RPS4[Arabidopsis thaliana] CAB50708.1

METSSISTVEDKPPQHQVFINFRGADLRRRFVSHLVTALKLNNINVFIDDYEDRGQPLDVLLKRIEESKIVLAIFSGNYTESVWCVRELEKIKDCTDEGTLVAIPIFYKLEPSTVRDLKGKFGDRFRSMAKGDERKKKWKEAFNLIPNIMGIIIDKKSVESEKVNEIVKAVKTALTGIPPEGSHNAVVGALGNSNAGTSSGDKKHETFGNEQRLKDLEEKLDRDKYKGTRIIGVVGMPGIGKTTLLKELYKTWQGKFSRHALIDQIRVKSKHLELDRLPQMLLGELSKLNHPHVDNLKDPYSQLHERKVLVVLDDVSKREQIDALREILDWIKEGKEGSRVVIATSDMSLTNGLVDDTYMVQNLNHRDSLQLFHYHAFIDDQANPQKKDFMKLSEGFVHYARGHPLALKVLGGELNKKSMDHWNSKMKKLAQSPSPNIVSVFQVSYDELTTAQKDAFLDIACFRSQDKDYVESLLASSDLGSAEAMSAVKSLTDKFLINTCDGRVEMHDLLYKFSREVDLKASNQDGSRQRRLWLHQHIIKGGIINVLQNKMKAANVRGIFLDLSEVEDETSLDRDHFINMGNLRYLKFYNSHCPQECKTNNKINIPDKLKLPLKEVRCLHWLKFPLETLPNDFNPINLVDLKLPYSEMEQLWEGDKDTPCLRWVDLNHSSKLCSLSGLSKAEKLQRLNLEGCTTLKAFPHDMKKMKMLAFLNLKGCTSLESLPEMNLISLKTLTLSGCSTFKEFPLISDNIETLYLDGTAISQLPMNMEKLQRLVVLNMKDCKMLEEIPGRVGELKALQELILSDCLNLKIFPEIDISFLNILLLDGTAIEVMPQLPSVQYLCLSRNAKISCLPVGISQLSQLKWLDLKYCTSLTSVPEFPPNLQCLDAHGCSSLKTVSKPLARIMPTEQNHSTFIFTNCENLEQAAKEEITSYAQRKCQLLSYARKRYNGGLVSESLFSTCFPGCEVPSWFCHETVGSELEVKLLPHWHDKKLAGIALCAVVSCLDPQDQVSRLSVTCTFKVKDEDKSWVAYTCPVGSWTRHGGGKDKIELDHVFIGYTSCPHTIKCHEEGNSDECNPTEASLKFTVTGGTSENGKYKVLKCGLSLVYAKDKDKNSALETKYDMLIGKSFQETSEGVDGRVKKTKGKYVMPVEKNFQETTEGVDGRVNKKKKTRMDNGRPKKKQRSGRDDNQTRMQVELQEGNINSVIMHTVKNF

>RPS2[Arabidopsis thaliana] AAM90858.1

MDFISSLIVGFAQVLCESMNMADRRGHNTDLRQAITDLETAIGDLKAIRDDLSLRIQQDDLEGRSCSNRAREWLSAVQATETKSASILVRFRRREQRTRMRRRCLGCFGCADYKLCNKVSATLKSIGELRERSEDIKTDGGSIQQTCREIPIKSVVGNTTMMEQVLGFLSEEEERGIIGVYGPGGVGKTTLMQSINNELITKGHQYDVLIWVQMSREFGECTIQQAVGAQLGLSWDEKDTGENRALKIYRALRQKRFLLLLDDVWEEIDLEKTGVPRPDRVNKCKMMFTTRSMALCSNMGAEYKLRVEFLEKKYAWELFCSKVGRKDLLESSSIRRLAEIIVSKCGGLPLALITLGGAMAHRETEEEWIHASEVLTRFPAEMKGMNYVFALLKFSYDNLESDLLRSCFLYCALFPEEHSIEIEQLVEYWVGEGFLTSSHGVNTIYKGYFLIGDLKAACLLETGDEKTQVKMHNVVRSFALWMASEQGTYKELILVEPNMGHTEAPKAENWRQALVISLIDNRIQTLPEKPICPKLTTLMLQRNSSLKKISTGFFMHMPILRVLDLSFTSITEIPLSIKYLVELCHLSMSGTKISILPQELGNLRKLKHLDLQRTQFLQTIPRDAICWLSKLEVLNLYYSYAGWELQSFGEDKVEELGFDDLEYLENLTTLGITVLSLETLKTLYEFGALHKHIQHLHIEECNGLLYFNLPSLTNHGRNLRRLSIRSCHDLEYLVTPIDVVENDWLPRLEVLTLHSLHKLSRVWRNPVSEDECLRNIRCINISHCNKLKNVSWVPKLPKLEVIDLFDCRELEELISEHESPSVEDPTLFPSLKTLKTRDLPELKSILPSRFSFQKVETLVITNCPKVKKLPFQETNMPRVYCEEKWWNALEKDEPNKELCYLPRFVPN
