# Supplementary material for: Overexpression of a novel peanut NBS‐LRR gene AhRRS5 enhances disease resistance to Ralstonia solanacearum in tobacco
Source: Plant Biotechnol J. 2016 Jul 26;15(1):39–55. doi: 10.1111/pbi.12589 (PMC5253469; doi:10.1111/pbi.12589)
Supplement: Supplementary file 7 — Data S5 qPCR analysis of relative transcript levels of defence marker genes in leaves of T2 AhRRS5‐OE‐3 lines compared to that in leaves of wild‐type tobacco plants. [file PBI-15-39-s004.docx]

Data S-5

**Figure S5.** qPCR analysis of relative transcript levels of defense marker genes in leaves of T_2_ *AhRRS5-OE-3* lines compared to that in leaves of wildtype tobacco plants. WT, untransgenic wildtype tobacco CB-1. The transcript levels of *NtH1N1*, *NtHSR201*, *NtHSR203*, *NtHSR515*, *NtPR-1a/c*, *NtPR3*, *NtPR4*, *NtNPR1*, *NtPR2*, *NtPR-1b*, *NtEFE26*, and *NtAsc6* were determined by quantitative real-time PCR. Relative transcript levels were normalized using the transcripts of *NtEF1ɑ*. Transcript levels of defense-related genes of wildtype tobacco plants were used as reference, which was set to a relative expression level of ‘1’. Error bars indicate the standard error; the experiments were repeated three times with at least three independent repetitions of the biological experiments. Asterisks indicate a significant difference (Student–Newman–Keuls test, **P <* 0.05 or ***P <* 0.01).

***AhRRS5-OE-3***

***WT***


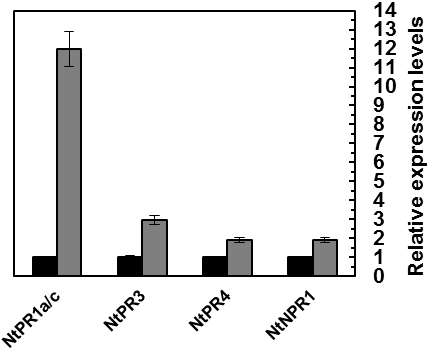

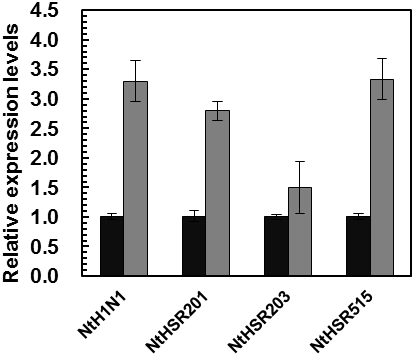


******

******

******

******

******

******

******

**HR responsive genes**

**SA responsive genes**


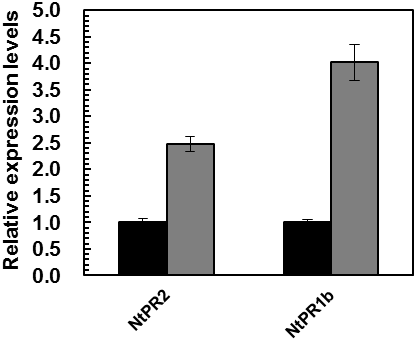

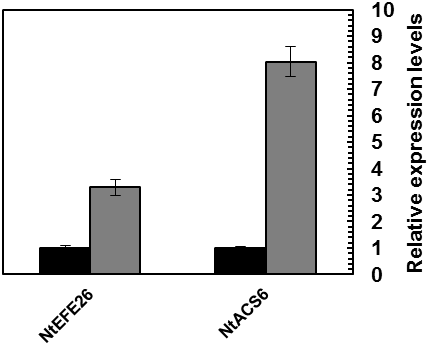


******

******

******

******

**JA responsive genes**

**ET responsive genes**
